# Proteomics-Led Multi-Omics Analysis Characterizes Candidate Molecular Features Accompanying Higher IMP Content in the Longissimus dorsi Muscle of New Jilin Black Pigs

**DOI:** 10.3390/foods15213950

**Published:** 2026-11-03

**Authors:** Zhidong Lv, Ruige Chu, Han Chu, Jianing Ma, Chunmei Yu, Tiantian Sun, Xingyao Pei, Liang Hong, Na Li, Lei Pu

**Affiliations:** 1 Tianjin Key Laboratory of Agricultural Animal Breeding and Healthy Husbandry, College of Animal Science and Veterinary Medicine, Tianjin Agricultural University, Tianjin 300392, China; 2 Jilin Academy of Agricultural Sciences (Northeast Agricultural Research Center of China), Changchun 130033, China

**Keywords:** Duroc × Landrace × Yorkshire, purine metabolism, nucleotide turnover, untargeted metabolomics, transcriptomics, pork quality

## Abstract

Inosine 5′-monophosphate (IMP) is an important umami-associated nucleotide in meat, yet whether New Jilin Black (NJ) and Duroc × Landrace × Yorkshire (DLY) pigs differ in longissimus dorsi muscle IMP content and which molecular features accompany any such difference remain unclear. Longissimus dorsi muscles from six intact male pigs per breed, selected at similar body weight but not matched for age, were analyzed using targeted IMP quantification and a proteomics-led multi-omics strategy. IMP quantification and untargeted metabolomics used six pigs per breed, whereas proteomic and transcriptomic analyses used four randomly selected pigs per breed. IMP content was higher in NJ than in DLY (1965.0 ± 134.0 vs. 1407.7 ± 115.1 mg/kg; *p* < 0.0001). Two proteins met proteome-wide FDR and fold-change criteria, whereas RPIA, GART, NT5C1A, and NUDT16 were retained as exploratory purine-related candidates. Metabolomics identified 279 FDR- and fold-change-supported differential features, including features annotated as hypoxanthine, xanthosine, and guanine with higher relative abundance and a feature annotated as adenylosuccinic acid with lower relative abundance in NJ. Corresponding transcripts showed the same NJ/DLY direction but were not significant after multiple-testing correction. These findings provide a phenotype-oriented, hypothesis-generating molecular context accompanying the higher measured IMP content in NJ and require independent targeted, functional, and sensory validation.

## 1. Introduction

Pork quality is a multidimensional concept encompassing sensory, technological, and nutritional attributes, with flavor, tenderness, meat color, and water-holding capacity representing important components of pork quality and consumer acceptance [1,2]. Duroc × Landrace × Yorkshire (DLY) pigs are a widely used commercial three-way crossbred population and represent one of the major commercial crossbred pig populations used for pork production in China [3,4,5]. Their widespread use in commercial production makes DLY pigs a practically relevant reference population for comparisons with Chinese indigenous or locally developed pigs. Compared with Western lean-type breeds or commercial crossbred pigs, many Chinese indigenous or black pig breeds exhibit breed-associated differences in meat quality and flavor-related characteristics. For example, Taoyuan Black pigs differ from Duroc pigs in meat quality and several flavor-related components, including flavor nucleotides, free amino acids, and fatty acids [6]. Likewise, comparisons between Chinese indigenous or black pigs and DLY pigs have revealed differences in carcass and meat-quality traits, metabolomic profiles, and flavor-related compounds [7,8,9]. Therefore, DLY pigs provide a commercially relevant benchmark for evaluating breed-associated differences in meat quality and flavor-related characteristics between Chinese indigenous or black pigs and commercial crossbred populations. Against this comparative background, omics-based approaches provide a useful framework for investigating molecular features associated with breed-related differences in pork quality.

New Jilin Black (NJ) pigs represent a regional black-pig genetic resource in Jilin Province, China, and are included in local efforts related to germplasm conservation and utilization and new-breed development [10]. As a locally important black-pig genetic resource, further characterization of its meat-quality-related biochemical and molecular features may provide useful information for understanding its phenotypic characteristics and supporting its germplasm evaluation and utilization. Previous omics-based studies in Chinese indigenous or black pigs have revealed breed-related molecular and metabolic differences associated with meat quality and flavor precursors [7,11]. However, whether comparable molecular and metabolic patterns occur in NJ remains unclear, as molecular evidence for this population is still limited. Therefore, comparison of NJ with a commercially relevant DLY benchmark provides an opportunity to characterize molecular and metabolic differences associated with pork quality and flavor-related traits in NJ.

Pork flavor is an important factor influencing consumer acceptance and is shaped by both volatile aroma compounds and non-volatile taste compounds. While volatile compounds primarily contribute to aroma, non-volatile water-soluble compounds, including free amino acids, 5′-nucleotides, taste-active peptides, organic acids, soluble sugars, and inorganic ions, contribute to the taste characteristics of meat [12]. Among these compounds, 5′-nucleotides are important components of the non-volatile taste profile, and IMP, AMP, and GMP are commonly examined in studies of pork taste-related chemistry [13,14]. In particular, inosine 5′-monophosphate (IMP) is widely recognized as an important umami-associated nucleotide in meat [14,15], and differences in IMP content have been reported among pig breeds [8,9]. Accordingly, IMP represents a relevant biochemical trait for investigating breed-related variation in non-volatile taste-related characteristics of pork. The contribution of IMP to perceived umami, however, depends on the broader chemical context of meat. IMP can interact synergistically with umami-active amino acids such as glutamate [16]. Accordingly, assessments based on taste-active compound concentrations, equivalent umami concentration (EUC), electronic tongue analysis, and sensory evaluation provide complementary information on umami-related taste properties [12,17]. Therefore, IMP content alone should be interpreted as reflecting IMP-related biochemical umami potential rather than as a direct measure of sensory umami intensity.

From a metabolic perspective, IMP in postmortem muscle is closely linked to purine nucleotide turnover and postmortem energy metabolism. Following slaughter, declining ATP availability, glycogen breakdown, and glycolytic activity contribute to changes in postmortem muscle metabolism and meat-quality development [18]. Metabolomic studies have further shown that purine-related metabolites change during postmortem aging and that these metabolic patterns may differ among breeds [19,20]. In pork, comparisons between Chinese indigenous or black pigs and commercial crossbred pigs have identified breed-related differences in purine metabolism [7], while studies involving indigenous and hybrid pigs have also reported differences in nucleotide- and flavor-related metabolites [11]. Evidence from other muscle systems has likewise linked IMP-related variation to purine metabolism, including processes involved in IMP synthesis and degradation [21,22]. Collectively, these findings support considering purine nucleotide turnover and postmortem energy metabolism when investigating breed-related differences in IMP content.

Multi-omics approaches provide complementary molecular information for investigating complex meat-quality traits by integrating changes at the transcript, protein, and metabolite levels [23]. Metabolomics is particularly suited to characterizing small-molecule changes associated with taste-related phenotypes, whereas proteomics directly measures protein abundance and can identify candidate enzymes and proteins involved in relevant metabolic pathways [23]. Importantly, protein abundance is shaped not only by transcript abundance but also by post-transcriptional and translational regulation and protein degradation [24]. Illustrating this potential transcript–protein discordance, a recent study of porcine longissimus dorsi muscle reported a weak but statistically significant correlation between mRNA and protein expression levels [25]. Proteomic studies in porcine longissimus dorsi muscle have further identified proteins associated with energy metabolism and other meat-quality-related metabolic processes [25,26], while an IMP-focused proteomic–metabolomic study in chicken muscle identified purine-metabolism-related proteins and metabolites associated with differences in IMP deposition [22]. Therefore, given the aim of characterizing the protein-level molecular context associated with breed-related differences in IMP content, proteomics was selected as the primary discovery layer; metabolomics was used to characterize the accompanying small-molecule context, whereas transcriptomics provided complementary transcript-level information.

Against this background, whether NJ and DLY pigs differ in IMP content and whether such differences are accompanied by corresponding molecular features remain insufficiently characterized, particularly at the protein and metabolite levels. We hypothesized that NJ and DLY pigs would differ in the IMP content of the longissimus dorsi muscle and that this phenotypic difference would be accompanied by differences in protein abundance and metabolite profiles, particularly in molecular features related to purine nucleotide turnover and postmortem energy metabolism. To examine this hypothesis, we quantified IMP content in the longissimus dorsi muscle of NJ and DLY pigs and applied a proteomics-led multi-omics strategy integrating proteomics, untargeted metabolomics, and complementary transcriptomic information to characterize candidate molecular features associated with breed-related differences in IMP content. A schematic overview of the experimental design and proteomics-led multi-omics workflow is provided in Figure S1.

## 2. Materials and Methods

### 2.1. Experimental Animals and Sample Collection

Experimental pigs were obtained from the Breeding Pig Farm of the Jilin Academy of Agricultural Sciences and were raised under the same management conditions. When the pigs reached a body weight of approximately 100 kg, six intact male NJ pigs and six intact male DLY pigs were randomly selected within each breed for the experiment, yielding a total of 12 animals. The six pigs within each breed were from different litters. Because NJ and DLY pigs differ in growth rate, the comparison was conducted at a similar target body weight rather than at the same chronological age. At slaughter, NJ and DLY pigs were 193.8 ± 6.6 d (185–201 d) and 158.0 ± 7.9 d (150–168 d) old, respectively, with body weights of 99.5 ± 3.4 and 101.0 ± 2.5 kg (mean ± SD, *n* = 6 per breed).

Throughout the experimental period, pigs of both breeds were maintained under the same feeding and management conditions. Each pig was housed individually in a pen of approximately 1.8 m^2^ equipped with an individual feeder and a nipple drinker. The pens had solid flooring and semi-open partitions. Ambient temperature was maintained at approximately 23 °C, with a light period of approximately 06:00–18:00. Feed was provided twice daily at approximately 07:00 and 14:00, with ad libitum access to feed and water. Both breeds received the same basal diet throughout the experimental period; its ingredient composition and analyzed nutrient levels are provided in Supplementary Table S1. Routine disinfection and immunization were conducted according to the standard management procedures of the farm.

Before slaughter, all pigs were fasted for 12 h with free access to water. All 12 animals were slaughtered on the same day, within the same slaughter batch, and at the same commercial abattoir under standardized procedures. Within 40 min postmortem, longissimus dorsi muscle samples were collected from the left side of each carcass. The subcutaneous fat layer at the sampling site was first removed, followed by trimming of visible surface fat and fascia. Muscle tissue was then collected along the anterior edge of the penultimate thoracic vertebra. Immediately after excision and trimming, the samples were snap-frozen in liquid nitrogen and transported to the laboratory on dry ice. Samples designated for targeted IMP determination were processed upon arrival at the laboratory. Samples designated for omics analyses were stored at −80 °C for approximately 72 h before being transported on dry ice to Novogene Co., Ltd. (Beijing, China) for subsequent analysis.

All six pigs per breed were included in targeted IMP determination and untargeted metabolomic analysis. For proteomic and transcriptomic analyses, four animals were randomly selected from the six animals within each breed. Random selection was used to reduce the potential for subjective selection bias when defining the omics subset. The same four animals within each breed were used for both proteomic and transcriptomic analyses, thereby maintaining matched biological samples across these two omics layers. The individual pig was considered the experimental unit for all biological comparisons.

Animal experimental and sample collection procedures were approved by Jilin Academy of Agricultural Sciences (approval no. JNK20240828-04).

### 2.2. Determination of IMP Content

IMP content in longissimus dorsi muscle was determined by high-performance liquid chromatography (HPLC) according to T/NAIA 003-2020 [27]. Frozen muscle samples were ground under liquid nitrogen before extraction. For each biological sample, two parallel 2.0 g portions were weighed into separate 50 mL centrifuge tubes and processed independently. Each portion was extracted by homogenization in three sequential steps using a total of 20 mL of pre-chilled 5% perchloric acid. The homogenates were centrifuged at 4000 rpm for 10 min at 4 °C. For each parallel portion, the supernatants obtained from the sequential extraction steps were pooled, adjusted to pH 6.5 with 2 mol/L sodium hydroxide, and brought to a final volume of 50 mL with water. The extracts were mixed thoroughly and filtered through a 0.45 μm membrane before HPLC analysis. The two parallel portions from each biological sample were independently extracted and analyzed, and the mean of the two independent determinations was used as the IMP content of each biological sample. A reagent blank was prepared and processed in parallel following the same procedure without muscle tissue.

Chromatographic separation was performed using an HPLC system equipped with a UV detector (Agilent Technologies, Santa Clara, CA, USA) and a C18 column (250 × 4.6 mm, 5 μm, Shimadzu Corporation, Kyoto, Japan) maintained at 25 °C. The triethylamine–phosphoric acid solution was prepared by adding 3.5 mL of phosphoric acid and 7.2 mL of triethylamine to 200 mL of water, adjusting the pH to 6.5 with triethylamine, and then diluting the solution to 1 L with water. The mobile phase consisted of the triethylamine–phosphoric acid solution and methanol at a ratio of 95:5 (*v*/*v*). The mobile phase was filtered through a 0.45 μm membrane and ultrasonically degassed before use. Isocratic elution was performed at a flow rate of 1.0 mL/min. The injection volume was 20 μL, and UV detection was performed at 254 nm. IMP was quantified by external-standard calibration based on chromatographic peak area and expressed as mg/kg of muscle tissue.

Method performance was evaluated in terms of linearity, recovery, intra-day precision, limit of detection (LOD), and limit of quantification (LOQ). Linearity was assessed using six IMP standard concentrations of 4, 8, 20, 40, 80, and 120 μg/mL. The calibration curve showed good linearity over the range of 4–120 μg/mL, with the regression equation y = 31,757.23x − 26,160.70 and a coefficient of determination (R^2^) of 0.9998, where x represents IMP concentration (μg/mL) and y represents chromatographic peak area. Recovery was evaluated in duplicate at fortification levels of 50, 750, and 3000 mg/kg, yielding mean recoveries of 98.2%, 96.8%, and 98.6%, respectively. Intra-day precision was evaluated using seven replicate measurements and yielded a relative standard deviation (RSD) of 1.87%. The LOD and LOQ were estimated as 3S and 10S, respectively, where S represents the standard deviation of 11 replicate measurements of a low-level fortified sample (50 mg/kg). The resulting LOD and LOQ were 2.4 and 8.0 mg/kg, respectively.

A certified 5′-inosinic acid reference material (certified purity, 94.3%; product code 72584-10mg; lot no. 23090189; TMstandard, Tan-Mo Technology Co., Ltd., Changzhou, Jiangsu, China) was used as the calibration standard.

### 2.3. Transcriptomic Analysis

Four longissimus dorsi samples from each group were used for transcriptomic analysis. After total RNA extraction, RNA integrity and quantity were assessed using an Agilent 2100 Bioanalyzer (Agilent Technologies, Santa Clara, CA, USA) The RNA integrity number (RIN) values of the eight samples ranged from 8.0 to 9.3. Qualified RNA samples were used for subsequent library construction. Briefly, mRNA with poly(A) tails was enriched using Oligo(dT) magnetic beads and randomly fragmented. The fragmented mRNA was then used as a template for cDNA synthesis using random oligonucleotide primers. Double-stranded cDNA was subjected to end repair, A-tailing, adapter ligation, fragment selection, and PCR amplification to generate sequencing libraries. All eight libraries were prepared within the same library-preparation batch. The libraries were initially quantified using a Qubit 2.0 Fluorometer (Life Technologies, Carlsbad, CA, USA), assessed for insert-size distribution using an Agilent 2100 Bioanalyzer, and further quantified by quantitative real-time PCR to determine the effective library concentration. Qualified libraries were sequenced on an Illumina platform to generate 150 bp paired-end reads.

Raw reads were processed using fastp v0.19.7 to obtain clean reads by removing adapter-containing reads, reads containing undetermined bases, and low-quality reads, defined as reads in which bases with Qphred ≤ 5 accounted for more than 50% of the read length. The proportions of bases with Phred quality scores ≥ 20 (Q20) and ≥ 30 (Q30), together with guanine–cytosine (GC) content of the clean reads were calculated, and all subsequent analyses were based on the clean reads. Across the eight samples, 6.88–7.62 Gb of clean sequencing data were obtained per sample, with Q30 values ranging from 95.37% to 97.35%. Clean reads were aligned to the pig reference genome *Sus scrofa* 11.1 (GCA_000003025.6) using HISAT2 v2.0.5, with total mapping rates of 92.13–94.01% and uniquely mapped rates of 86.41–89.62%. The number of reads mapped to each gene was calculated using featureCounts v1.5.0-p3. Gene expression levels were estimated as fragments per kilobase of transcript per million mapped reads (FPKM) for expression profiling, whereas read counts were used for differential expression analysis. Differential expression analysis was performed using DESeq2 v1.20.0, and *p* values were adjusted using the Benjamini–Hochberg method to control the false discovery rate. Detailed sample-level RNA quality, sequencing, and mapping statistics are provided in Supplementary Table S2, and complete gene-level differential-expression results are provided in Supplementary Table S3.

### 2.4. Proteomic Analysis

Four longissimus dorsi samples from each group were used for proteomic analysis. Frozen muscle samples were processed under low-temperature conditions for protein extraction, quantification, and quality assessment. Equal amounts of protein from each sample were subjected to reduction, alkylation, and trypsin digestion to obtain peptide mixtures. The resulting peptide samples were desalted, dried, reconstituted, and analyzed using a Vanquish Neo–Astral liquid chromatography–mass spectrometry platform with data-independent acquisition (DIA). All eight samples were analyzed within the same LC–MS analytical batch, and the injection order was randomized across samples.

Raw mass spectrometry data were saved in .raw format and imported into DIA-NN v1.8.1 for database searching, peptide identification, and protein-level quantification. The pig reference protein database 2748279_Sus_scrofa.Sscrofa11.new.fasta was used for database searching. Trypsin was selected as the digestion enzyme, and the maximum number of missed cleavages was set to 2. Carbamidomethylation of cysteine was set as a fixed modification, and N-terminal methionine excision was set as a variable modification. Peptide- and protein-level identifications were both controlled at a false discovery rate (FDR) of <0.01. Protein abundance values were normalized using sample-wise SUMmax normalization. Briefly, the sum of all protein quantitative values was calculated for each sample, and the ratio of the total protein abundance of each sample to the maximum total abundance among all samples was used as the sample-specific normalization factor. Each original protein quantitative value was then divided by the corresponding normalization factor. Zero values represented non-detected proteins and were not imputed. The normalized protein abundance values were log_2_-transformed before subsequent statistical analysis.

The normalized protein quantification matrix was used for differential-abundance and functional analyses. Of the 4001 protein entries in the processed matrix, 3995 proteins with at least two quantified observations in each group were retained for continuous-abundance analysis using the limma empirical Bayes model. The remaining six proteins showed group-specific detection patterns and were excluded from the continuous-abundance model because zero values were not imputed and reliable finite fold-change estimates could not be obtained. For the 3995 tested proteins, NJ/DLY log_2_ fold changes, 95% confidence intervals, raw *p* values, and Benjamini–Hochberg (BH)-adjusted *q* values were calculated. Proteins satisfying both BH-adjusted *q* ≤ 0.05 and |log_2_FC| ≥ log_2_(1.5) were considered FDR-supported differentially abundant proteins. Proteins satisfying raw *p* < 0.05 and |log_2_FC| ≥ log_2_(1.5) were additionally retained as a nominal exploratory protein set for descriptive functional analyses.

Principal component analysis (PCA) was performed on the scaled log_2_-transformed protein abundance matrix, and pairwise Pearson correlation coefficients were calculated to assess overall similarity among samples. Hierarchical clustering was performed using row-wise Z-score-standardized protein abundances, Euclidean distance, and complete-linkage clustering.

The nominal exploratory protein set was subjected to Gene Ontology (GO) functional annotation and Kyoto Encyclopedia of Genes and Genomes (KEGG) pathway over-representation analysis for descriptive functional characterization. All proteins entering the corresponding differential-abundance analysis were used as the background set. Enrichment *p* values were adjusted for multiple testing using the Benjamini–Hochberg method, and BH-adjusted *q* values were reported. Candidate proteins associated with purine nucleotide metabolism and nucleotide precursor/turnover processes, energy metabolism, and amino acid metabolism/peptide processing were subsequently prioritized in relation to the IMP phenotype for exploratory cross-omics integration. Phenotype-oriented proteins that did not meet the proteome-wide BH-adjusted significance criterion were classified as exploratory candidates.

### 2.5. Untargeted Metabolomic Analysis

Six longissimus dorsi samples from each group were used for untargeted metabolomic analysis. Briefly, frozen muscle samples were ground in liquid nitrogen, and 100 mg of tissue powder was transferred into a microcentrifuge tube. Then, 500 μL of 80% aqueous methanol was added for metabolite extraction. The mixture was vortexed, incubated on ice for 5 min, and centrifuged at 15,000× *g* for 20 min at 4 °C. An aliquot of the supernatant was diluted with LC–MS-grade water to a final methanol concentration of 53%, followed by centrifugation at 15,000× *g* for 20 min at 4 °C. The resulting supernatant was collected for LC–MS/MS-based metabolomic analysis. Quality control (QC) samples were prepared by pooling equal aliquots from the samples included in the analytical run and were used to monitor the stability of the LC–MS system. Pooled QC samples were analyzed before, during, and after the biological sample sequence. Blank samples were prepared using 53% aqueous methanol instead of biological samples and were processed using the same procedure to remove background ions. Biological samples were analyzed within the same LC–MS/MS analytical sequence using a randomized injection order. No internal standards were used in the untargeted metabolomic analysis.

Metabolomic data were acquired using a Vanquish ultra-high-performance liquid chromatography (UHPLC) system coupled to a Q Exactive™ HF mass spectrometer (Thermo Fisher Scientific, Bremen, Germany). Chromatographic separation was performed on a Hypersil Gold C18 column (100 × 2.1 mm, 1.9 μm; Thermo Fisher Scientific, Waltham, MA, USA) at a column temperature of 40 °C and a flow rate of 0.2 mL/min. Mobile phase A was 0.1% formic acid in water, and mobile phase B was methanol. The gradient elution program was as follows: 0–1.5 min, 98% A and 2% B; 3 min, 15% A and 85% B; 10 min, 0% A and 100% B; and 10.1–12 min, 98% A and 2% B. Mass spectrometric detection was performed using an electrospray ionization (ESI) source in both positive- and negative-ion modes. The scan range was m/z 100–1500. The ESI parameters were set as follows: spray voltage, 3.5 kV; sheath gas flow rate, 35 psi; auxiliary gas flow rate, 10 L/min; capillary temperature, 320 °C; S-lens RF level, 60; and auxiliary gas heater temperature, 350 °C. Data-dependent acquisition was used for MS/MS analysis.

Raw mass spectrometry data were converted to mzXML format using ProteoWizard (v3.0) and subsequently processed using XCMS (v3.2) for peak extraction, peak alignment, retention-time correction, and peak-area quantification. Features with a missing rate > 50% within a group were removed during data preprocessing, and zero values in the retained feature matrix were imputed using the K-nearest neighbors (KNN) method. Peak-area values were normalized to the total peak area within each sample. Metabolite features with a coefficient of variation (CV) ≥ 30% across pooled QC samples were excluded from subsequent analysis. Metabolite annotation was performed by matching molecular features against the Novogene in-house metabolite database using mass-to-charge ratio, adduct information, and high-quality MS/MS spectral information. Vendor-defined annotation confidence was categorized as Level 1, Level 2, or Level 3: Level 1 indicated matches in MS1, MS/MS, and retention time; Level 2 indicated matches in MS1 and MS/MS; and Level 3 indicated an MS1-level match. After QC filtering, a total of 1857 ion-mode-specific metabolite features were retained for subsequent analysis, including 678 features in negative-ion mode and 1179 features in positive-ion mode. Among the retained features, the mean and median QC CVs were 9.84% and 7.42%, respectively, in negative-ion mode, and 10.20% and 7.92%, respectively, in positive-ion mode. Features with QC CV ≤ 20% accounted for 87.9% and 84.9% of the retained features in negative- and positive-ion modes, respectively. The pooled QC samples clustered tightly in PCA, and pairwise Pearson correlation coefficients among the QC samples were 0.994 in both ionization modes, indicating good analytical stability and reproducibility (Figure S2). Metabolite annotations were further referenced against the Kyoto Encyclopedia of Genes and Genomes (KEGG), Human Metabolome Database (HMDB), and LIPID MAPS databases.

PCA was used as the primary unsupervised multivariate analysis to visualize the overall distribution of the metabolomic profiles. Partial least squares-discriminant analysis (PLS-DA) was performed separately for the positive- and negative-ion datasets using metaX as an exploratory supervised analysis. Before model construction, normalized relative metabolite intensities were log_2_-transformed, mean-centered, and scaled to unit variance (UV scaling). PLS-DA models were constructed using two components and evaluated using seven-fold cross-validation and 200 permutation tests. Variable importance in projection (VIP) scores were retained as complementary multivariate information rather than being used as the primary criterion for statistical significance.

### 2.6. Bioinformatic and Cross-Omics Integration Analysis

Before cross-omics integration, each omics dataset was independently subjected to the platform-specific quality-control, filtering, and normalization procedures described above. These included RNA quality and sequencing/mapping quality assessment for transcriptomics; identification FDR control, abundance filtering, and normalization for proteomics; and blank-based background removal, pooled-QC assessment, QC-CV filtering, missing-value handling, and normalization for metabolomics. Untargeted metabolomics included six pigs per group, whereas the transcriptomic and proteomic datasets were derived from the same four pigs per group, as described in Section 2.1. The transcriptomic libraries and proteomic samples were processed within single platform-specific batches, and the metabolomic samples were analyzed within the same LC–MS/MS analytical sequence. No within-platform batch correction was applied. Cross-omics integration was performed using QC-filtered group-level results and functional annotations rather than by directly concatenating raw abundance matrices across platforms.

After the individual proteomic, metabolomic, and transcriptomic analyses, cross-omics integration was performed using targeted IMP content as the phenotypic context. For pathway-level integration, KEGG annotation results from the nominal exploratory protein set were compared with those from the FDR-supported metabolite feature sets detected in positive- and negative-ion modes. Shared KEGG pathway annotations among these datasets were identified using the jvenn online tool, and the numbers of molecular features mapped to the shared pathway annotations were summarized using a matrix visualization.

For purine-related candidates prioritized in relation to the IMP phenotype, NJ/DLY log_2_FC estimates and 95% confidence intervals for representative proteins and annotated metabolite features were compared at the group level. For transcriptome–proteome comparison of the four purine-related candidate proteins (RPIA, GART, NT5C1A, and NUDT16), the corresponding transcript-level NJ/DLY log_2_FC values and Benjamini–Hochberg-adjusted *p* values were obtained from the complete DESeq2 results and compared with the protein-level log_2_FC estimates to assess concordance in the direction of change between the two omics layers.

The targeted IMP phenotype, purine-related candidate proteins, representative annotated metabolite features, and corresponding transcript-level results were subsequently integrated to construct a proposed molecular framework summarizing molecular features associated with the higher IMP content observed in NJ longissimus dorsi muscle.

Volcano plots for the proteomic and metabolomic datasets were generated using the BioLadder platform, whereas heatmaps were generated using the NovoMagic cloud platform (Novogene Co., Ltd., Beijing, China). The proteomic GO enrichment plot was generated using the SRplot online platform. Shared KEGG pathway annotations were identified using the jvenn online tool, and the shared-pathway matrix plot was generated using RAWGraphs 2.0. Group-level log_2_FC estimates and their 95% confidence intervals for representative proteins and metabolite features, as well as transcript–protein log_2_FC comparisons, were visualized using GraphPad Prism v11.0.2 (GraphPad Software, Boston, MA, USA). All figures were further optimized and arranged using Adobe Illustrator v29.5.1 (Adobe Inc., San Jose, CA, USA).

### 2.7. Statistical Analysis

The individual pig was considered the experimental unit. Targeted IMP content was analyzed using IBM SPSS Statistics v27.0.1.0 (IBM Corp., Armonk, NY, USA). Differences in IMP content between the NJ and DLY groups were evaluated using a two-sided independent-samples Student’s t-test. Data are presented as mean ± standard deviation (SD). The between-group mean difference and Hedges’ g were calculated and reported with their corresponding 95% confidence intervals (CIs). A two-sided *p* < 0.05 was considered statistically significant for the targeted IMP comparison. IMP plots and basic statistical graphics were generated using GraphPad Prism v11.0.2 (GraphPad Software, Boston, MA, USA).

For transcriptomic data, differential expression analysis was performed using DESeq2 v1.20.0 based on gene-level read counts. *p* values were adjusted using the BH method to control the FDR, and genes with an adjusted *p* ≤ 0.05 and |log_2_FC| ≥ 1 were considered differentially expressed genes.

For proteomic data, normalized and log_2_-transformed protein abundance values were analyzed using the limma empirical Bayes framework. For the 3995 proteins included in the differential-abundance analysis, NJ/DLY log_2_ fold changes, 95% confidence intervals (CIs), raw *p* values, and Benjamini–Hochberg-adjusted *q* values were calculated. BH correction was applied across all 3995 tested proteins. Proteins satisfying both BH-adjusted *q* ≤ 0.05 and |log_2_FC| ≥ log_2_(1.5) were considered FDR-supported differentially abundant proteins. Proteins satisfying raw *p* < 0.05 and |log_2_FC| ≥ log_2_(1.5) were additionally retained as a nominal exploratory protein set.

For untargeted metabolomic data, normalized relative-abundance values were log_2_-transformed and analyzed separately for the positive- and negative-ion datasets using the limma empirical Bayes framework. NJ/DLY log_2_FCs, 95% CIs, raw *p* values, and BH-adjusted *q* values were calculated for each metabolite feature. BH correction was applied jointly across all 1857 ion-mode-specific metabolite features. Metabolite features satisfying both BH-adjusted *q* ≤ 0.05 and |log_2_FC| ≥ log_2_(1.5) were considered FDR-supported differentially abundant features. Statistical significance of metabolite features was determined by the BH-adjusted *q*-value and fold-change criteria described above, whereas VIP scores from the PLS-DA models were retained as complementary multivariate descriptors.

## 3. Results

### 3.1. Higher IMP Content in the Longissimus Dorsi Muscle of NJ Pigs

**Figure 1:**
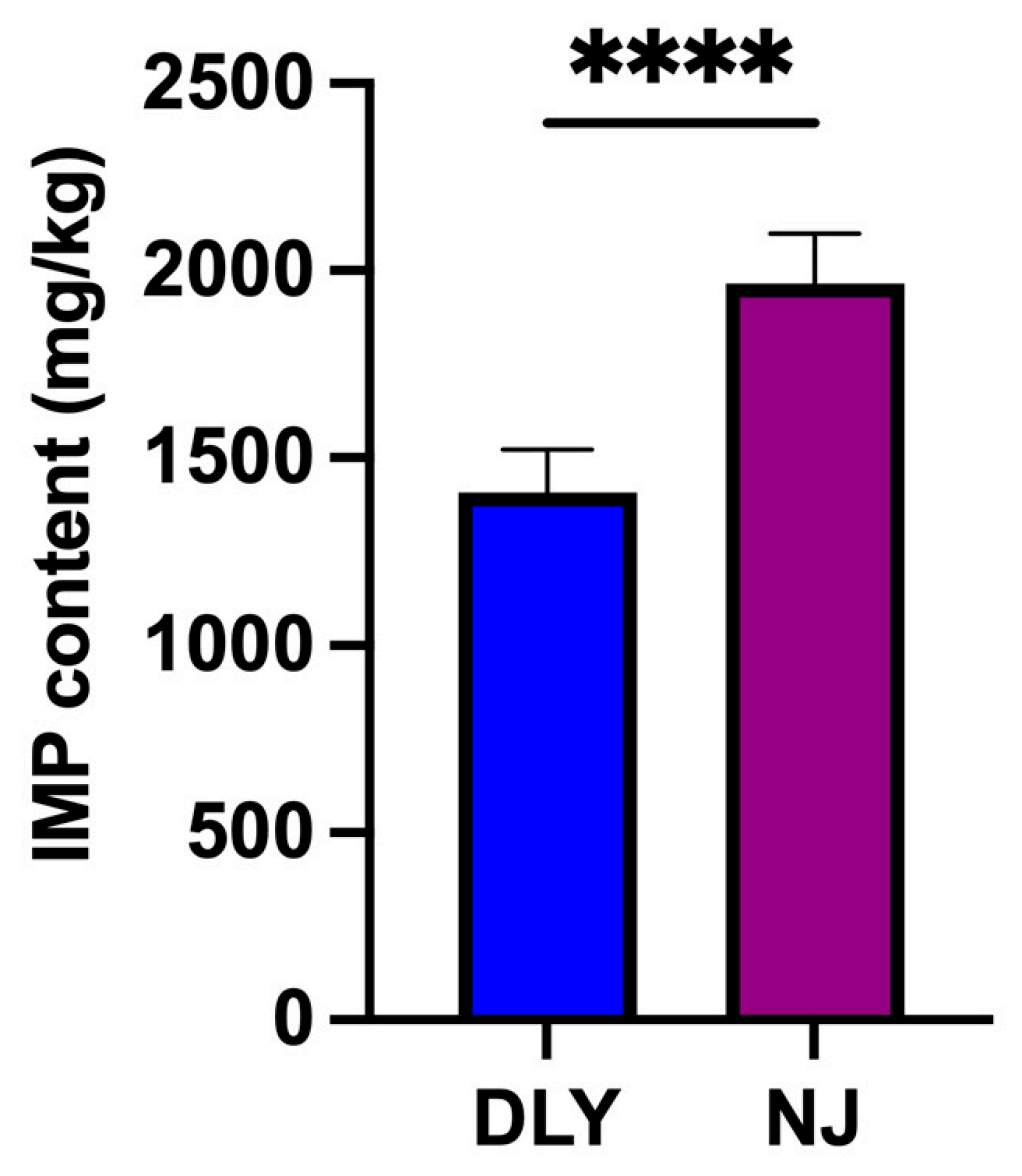
IMP content in the longissimus dorsi muscle of NJ and DLY pigs. Values are presented as mean ± SD (*n* = 6 per group). **** *p* < 0.0001, based on a two-sided independent-samples Student’s t-test. IMP, inosine 5′-monophosphate; NJ, New Jilin Black pigs; DLY, Duroc × Landrace × Yorkshire pigs.

Targeted HPLC quantification showed that IMP content in the longissimus dorsi muscle was significantly higher in NJ pigs than in DLY pigs (1965.0 ± 134.0 vs. 1407.7 ± 115.1 mg/kg, respectively; *p* < 0.0001; Table 1 and Figure 1). The estimated between-group mean difference was 557.3 mg/kg (95% CI, 396.6–718.0 mg/kg), with a Hedges’ g of 4.12 (95% CI, 2.03–6.15). The higher targeted IMP content observed in NJ was used as the core phenotypic anchor for the subsequent proteomic, untargeted metabolomic, and cross-omics analyses.

### 3.2. Global Proteomic Patterns in the Longissimus Dorsi Muscle of NJ and DLY Pigs

**Figure 2:**
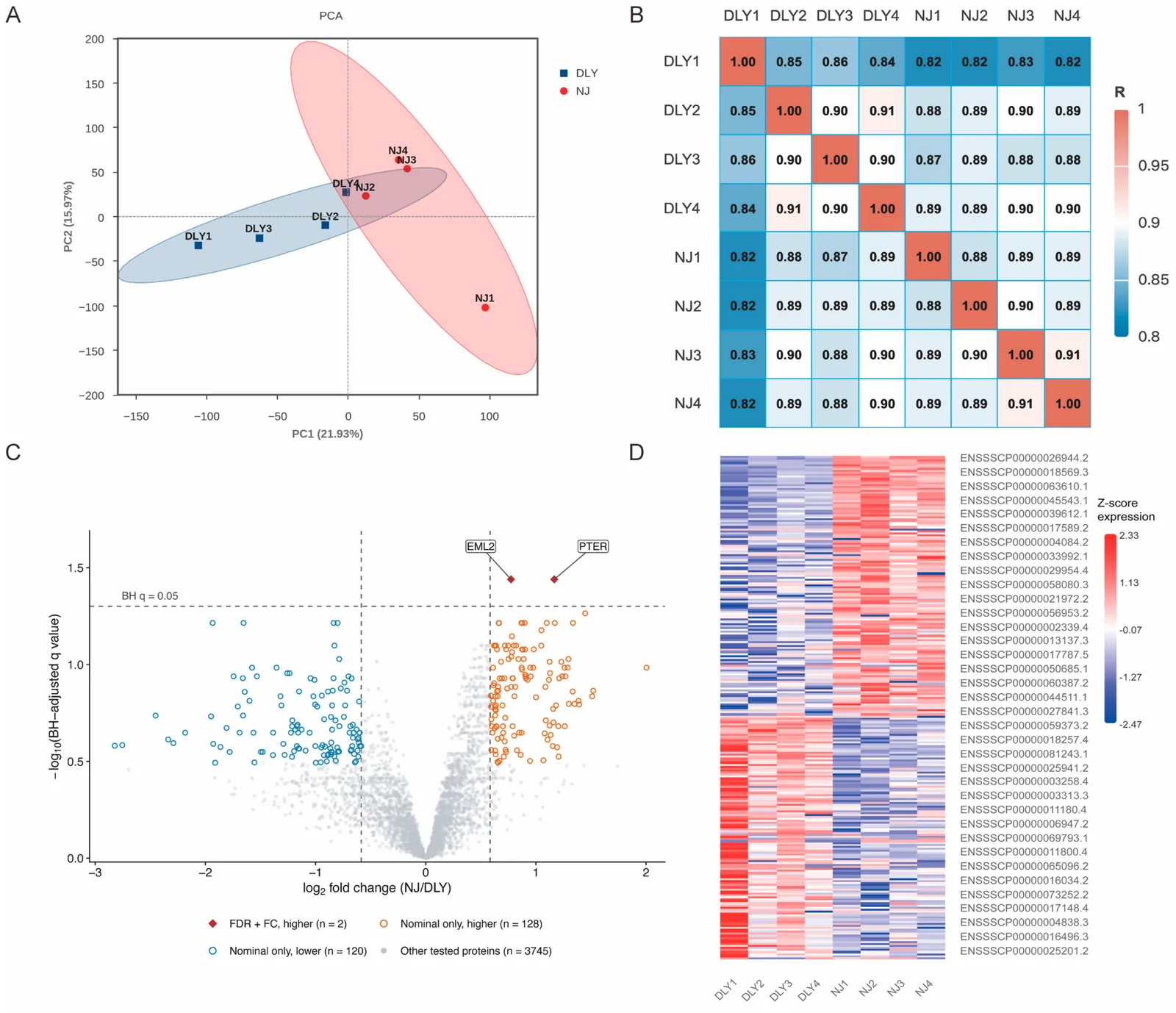
Global proteomic patterns in the longissimus dorsi muscle of NJ and DLY pigs. (**A**) Principal component analysis (PCA) of protein abundance profiles from four biological replicates per group. PC1 and PC2 explained 21.93% and 15.97% of the total variance, respectively. Ellipses represent the 95% confidence regions for each group. (**B**) Pairwise Pearson correlation matrix of the eight proteomic samples. Values within the matrix indicate Pearson correlation coefficients (r). (**C**) Volcano plot of protein abundance differences between NJ and DLY pigs based on the limma empirical Bayes analysis. The x-axis represents log_2_ fold change (NJ/DLY), and the y-axis represents −log_10_ (BH-adjusted *q* value). Vertical dashed lines indicate |log_2_ FC| = log_2_ (1.5), and the horizontal dashed line indicates *q* = 0.05. Red diamonds indicate proteins meeting both the FDR criterion of *q* ≤ 0.05 and the fold-change criterion (*n* = 2). Orange and blue circles indicate proteins meeting the nominal exploratory screening criteria (raw *p* < 0.05 and |log_2_ FC| ≥ log_2_ (1.5)) but not the FDR criterion, with higher (*n* = 128) and lower (*n* = 120) abundance in NJ, respectively. Other tested proteins are shown in light blue (*n* = 3745). (**D**) Hierarchical clustering heatmap of the 250 proteins meeting the nominal exploratory screening criteria. Columns represent individual samples and rows represent proteins. Relative protein abundance is displayed as row-wise Z-scores. Hierarchical clustering was performed using Euclidean distance and complete linkage. NJ, New Jilin Black pigs; DLY, Duroc × Landrace × Yorkshire pigs; BH, Benjamini–Hochberg; FDR, false discovery rate; FC, fold change.

Proteomic profiling was performed using four biological replicates per group. PCA based on protein abundance profiles showed partial separation between NJ and DLY samples, with PC1 and PC2 explaining 21.93% and 15.97% of the total variance, respectively (Figure 2A). Some overlap between the two groups and dispersion among NJ samples along PC2 were observed. Pairwise Pearson correlation coefficients among the eight proteomic samples ranged from 0.82 to 0.91 (Figure 2B).

Of the 4001 protein entries in the processed abundance matrix, 3995 met the criterion of at least two quantified observations per group and were included in the limma empirical Bayes analysis. The remaining six proteins showed group-specific detection and were not included in the continuous-abundance model. Among the 3995 tested proteins, 621 had a raw *p* < 0.05, 583 met the fold-change criterion of |log_2_FC| ≥ log_2_(1.5), and 250 met both nominal exploratory screening criteria. Of these 250 proteins, 130 showed higher abundance and 120 showed lower abundance in NJ than in DLY (Figure 2C). Following BH correction across all 3995 tested proteins, only phosphotriesterase-related protein (PTER) and echinoderm microtubule-associated protein-like 2 (EML2) met both the FDR criterion of *q* ≤ 0.05 and the fold-change criterion. PTER showed higher abundance in NJ (log_2_FC = 1.17, 95% CI, 0.85–1.49; raw *p* = 1.49 × 10^−5^; *q* = 0.036), as did EML2 (log_2_FC = 0.77, 95% CI, 0.56–0.99; raw *p* = 1.82 × 10^−5^; *q* = 0.036). Complete protein-level differential-abundance statistics, including log_2_FC estimates, 95% confidence intervals, raw *p* values, and BH-adjusted *q* values, are provided in Supplementary Table S4.

Hierarchical clustering of the 250 proteins meeting the nominal exploratory screening criteria was used to visualize their relative abundance patterns across individual NJ and DLY samples (Figure 2D). Because these proteins had been preselected on the basis of between-group abundance differences, the clustering heatmap was interpreted descriptively rather than as independent evidence of group separation. These 250 proteins were subsequently retained as an exploratory protein set for functional annotation.

### 3.3. Functional Annotation of the Exploratory Protein Set and Prioritization of IMP-Centered Candidate Proteins

**Figure 3:**
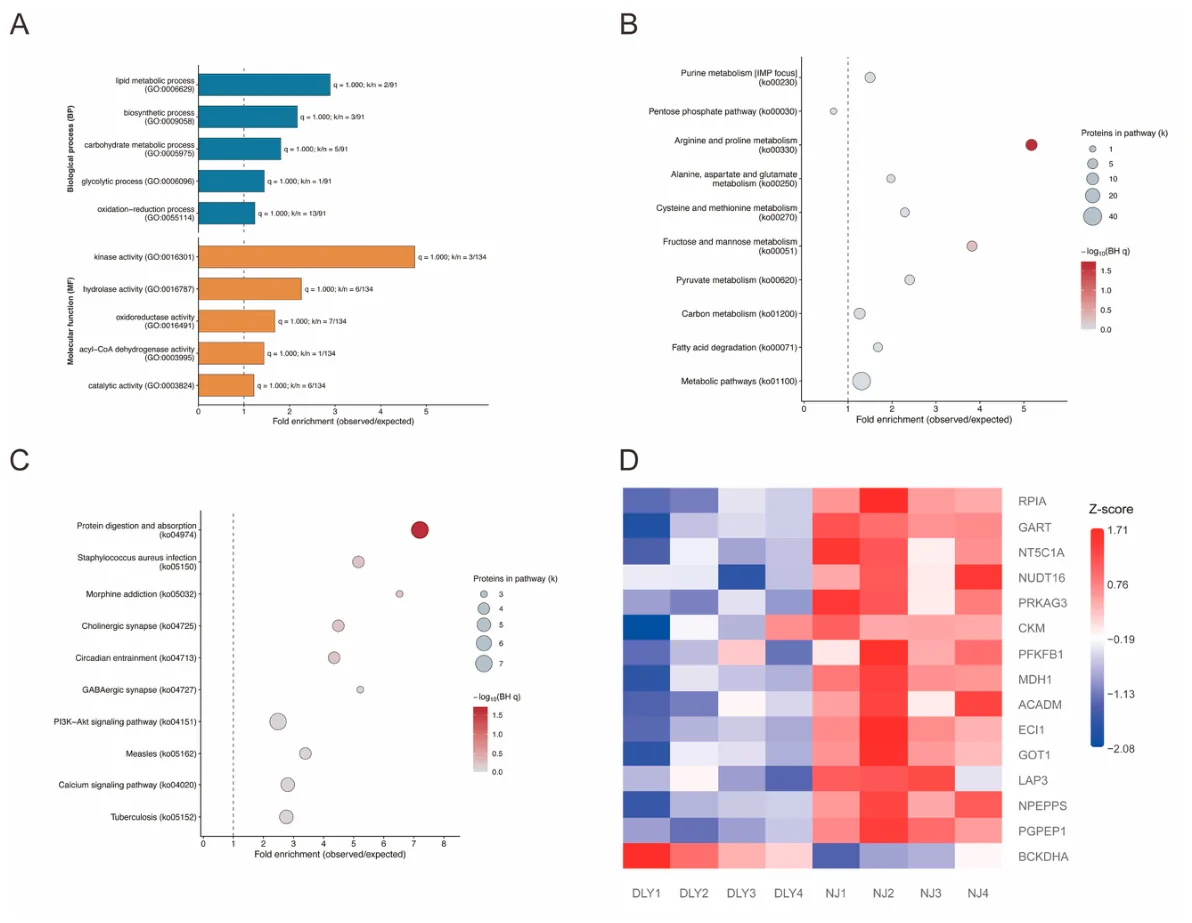
Functional Annotation of the Exploratory Protein Set and Prioritization of IMP-Centered Candidate Proteins. (**A**) Gene Ontology (GO) over-representation analysis of the 250 proteins meeting the nominal exploratory criteria of raw *p* < 0.05 and |log_2_FC| ≥ log_2_(1.5). (**B**) Kyoto Encyclopedia of Genes and Genomes (KEGG) over-representation analysis of the 130 exploratory proteins showing higher abundance in NJ. (**C**) KEGG over-representation analysis of the 120 exploratory proteins showing lower abundance in NJ. KEGG pathway visualization was based on multiple-testing-adjusted enrichment statistics. (**D**) Hierarchical clustering heatmap of 15 selected exploratory candidate proteins related to IMP-centered purine nucleotide metabolism, energy metabolism, and amino acid metabolism/peptide processing. Colors represent Z-score-normalized protein abundance across individual samples. All 15 proteins met the nominal exploratory criteria but did not meet the proteome-wide Benjamini–Hochberg-adjusted *q* ≤ 0.05 threshold. log_2_FC represents the NJ/DLY protein abundance ratio. IMP, inosine 5′-monophosphate; NJ, New Jilin Black pigs; DLY, Duroc × Landrace × Yorkshire pigs.

To further characterize the functional context of the exploratory protein abundance differences between NJ and DLY longissimus dorsi muscles, GO over-representation analysis was performed on the 250 proteins meeting the nominal exploratory screening criteria. Representative biological-process terms included lipid metabolic process, biosynthetic process, carbohydrate metabolic process, glycolytic process, and oxidation–reduction process, whereas representative molecular-function terms included kinase activity, hydrolase activity, oxidoreductase activity, acyl-CoA dehydrogenase activity, and catalytic activity (Figure 3A). Although the displayed terms showed varying degrees of fold enrichment, none remained significant after Benjamini–Hochberg correction (all BH-adjusted *q* = 1.000). Therefore, the GO results were interpreted as descriptive functional tendencies within the exploratory protein set rather than as FDR-supported enrichment.

Separate KEGG over-representation analyses were performed for the exploratory proteins showing higher (*n* = 130) and lower (*n* = 120) abundance in NJ. Among the representative pathways displayed for proteins with higher abundance in NJ, arginine and proline metabolism showed a relatively prominent enrichment signal, while proteins were also mapped to alanine, aspartate and glutamate metabolism, cysteine and methionine metabolism, fructose and mannose metabolism, pyruvate metabolism, carbon metabolism, fatty acid degradation, metabolic pathways, the pentose phosphate pathway, and purine metabolism (Figure 3B). Proteins with lower abundance in NJ were represented in pathways including protein digestion and absorption, PI3K–Akt signaling pathway, calcium signaling pathway, and several other signaling- or disease-associated KEGG categories (Figure 3C). The KEGG pathway plots were based on multiple-testing-adjusted enrichment statistics.

Among the 250 proteins, five were directly annotated to purine metabolism in KEGG: GART, NT5C1A, NUDT16, POLR1C, and POLR2D. GART showed nominally higher abundance in NJ (log_2_FC = 1.049, 95% CI: 0.596–1.502, raw *p* = 5.10 × 10^−4^, BH-adjusted *q* = 0.0798), as did NT5C1A (log_2_FC = 0.874, 95% CI: 0.410–1.337, raw *p* = 0.00203, *q* = 0.1061) and NUDT16 (log_2_FC = 0.606, 95% CI: 0.124–1.088, raw *p* = 0.0191, *q* = 0.2246). POLR1C also showed nominally higher abundance in NJ (log_2_FC = 1.106, 95% CI: 0.187–2.025, raw *p* = 0.0240, *q* = 0.2440), whereas POLR2D showed nominally lower abundance (log_2_FC = −1.223, 95% CI: −2.179 to −0.268, raw *p* = 0.0200, *q* = 0.2246). Among these five proteins, GART, NT5C1A, and NUDT16 were retained for IMP-centered exploratory interpretation because their individual functional annotations were more directly related to purine nucleotide biosynthesis or nucleotide hydrolysis and metabolism. Specifically, GART was annotated with phosphoribosylamine–glycine ligase activity and purine nucleobase biosynthetic process, NT5C1A with 5′-nucleotidase activity and nucleotide metabolic process, and NUDT16 with hydrolase activity. In contrast, the individual functional annotations of POLR1C and POLR2D were primarily related to DNA-directed RNA polymerase activity; therefore, they were not further prioritized as IMP-centered candidate proteins.

Although RPIA was not directly annotated to purine metabolism in KEGG, it showed nominally higher abundance in NJ (log_2_FC = 0.842, 95% CI: 0.432–1.251, raw *p* = 0.00116, BH-adjusted *q* = 0.0937). RPIA was annotated to the pentose phosphate pathway and carbon metabolism in KEGG and to ribose-5-phosphate isomerase activity in GO. Because ribose-5-phosphate provides a precursor-supply context for 5-phosphoribosyl-1-pyrophosphate (PRPP) formation, and PRPP is a basic substrate for de novo purine nucleotide biosynthesis [28,29], RPIA was additionally retained as an exploratory candidate related to the precursor-supply context of purine nucleotide biosynthesis. Accordingly, RPIA, GART, NT5C1A, and NUDT16 were retained as exploratory candidate proteins related to IMP-centered purine nucleotide metabolism for subsequent analyses (Figure 3D).

Given that postmortem IMP formation and turnover are closely associated with ATP degradation and AMP-related nucleotide metabolism during the conversion of muscle to meat [30], energy metabolism-related proteins were further examined. PRKAG3 (log_2_FC = 0.683, 95% CI: 0.344–1.023, raw *p* = 0.00131, BH-adjusted *q* = 0.0968), CKM (log_2_FC = 0.791, 95% CI: 0.338–1.243, raw *p* = 0.00324, *q* = 0.1178), PFKFB1 (log_2_FC = 0.596, 95% CI: 0.160–1.032, raw *p* = 0.0127, *q* = 0.1916), MDH1 (log_2_FC = 0.628, 95% CI: 0.357–0.900, raw *p* = 5.12 × 10^−4^, *q* = 0.0798), ACADM (log_2_FC = 0.621, 95% CI: 0.256–0.985, raw *p* = 0.00378, *q* = 0.1248), and ECI1 (log_2_FC = 1.051, 95% CI: 0.636–1.466, raw *p* = 2.66 × 10^−4^, *q* = 0.0665) all showed nominally higher abundance in NJ (Figure 3D). Based on functional annotation, PRKAG3 was mapped to the AMPK signaling pathway, CKM to phosphocreatine-related energy metabolism, PFKFB1 to glycolytic regulation, MDH1 to carbon and oxidation–reduction metabolism, and ACADM and ECI1 to fatty acid degradation. These proteins do not directly catalyze IMP formation; rather, their abundance differences were interpreted as complementary molecular features reflecting differences in the energy metabolism-related background between NJ and DLY muscles.

Candidate proteins related to amino acid metabolism and peptide processing were also examined. GOT1 showed nominally higher abundance in NJ (log_2_FC = 0.871, 95% CI: 0.413–1.329, raw *p* = 0.00190, BH-adjusted *q* = 0.1037), as did LAP3 (log_2_FC = 0.592, 95% CI: 0.217–0.967, raw *p* = 0.00591, *q* = 0.1446), NPEPPS (log_2_FC = 0.621, 95% CI: 0.345–0.896, raw *p* = 6.17 × 10^−4^, *q* = 0.0798), and PGPEP1 (log_2_FC = 0.638, 95% CI: 0.363–0.914, raw *p* = 5.07 × 10^−4^, *q* = 0.0798), whereas BCKDHA showed nominally lower abundance in NJ (log_2_FC = −0.732, 95% CI: −1.229 to −0.234, raw *p* = 0.00875, *q* = 0.1624) (Figure 3D). GOT1 was mapped to several amino acid-related KEGG pathways, including alanine, aspartate and glutamate metabolism and arginine and proline metabolism, supporting its relevance to amino acid conversion and carbon-skeleton metabolism. LAP3 and NPEPPS were associated with peptidase activity or proteolysis, whereas PGPEP1 was retained based on its annotation as pyroglutamyl-peptidase I. BCKDHA was annotated to valine, leucine and isoleucine degradation. These proteins do not directly participate in IMP formation and were therefore interpreted as complementary molecular features reflecting differences in amino acid conversion and peptide-processing processes that may be relevant to the non-volatile taste precursor background, rather than as direct determinants of IMP accumulation or sensory umami intensity.

Importantly, all 15 candidate proteins displayed in Figure 3D met the nominal exploratory criteria of raw *p* < 0.05 and |log_2_FC| ≥ log_2_(1.5), but none met the proteome-wide BH-adjusted *q* ≤ 0.05 threshold. They were therefore interpreted as exploratory candidate molecular features rather than as FDR-supported differentially abundant proteins. Overall, RPIA, GART, NT5C1A, and NUDT16 were retained as the principal exploratory candidates related to IMP-centered purine nucleotide metabolism, whereas the energy metabolism-related and amino acid metabolism/peptide-processing proteins provided complementary metabolic contexts for subsequent multi-omics analyses.

### 3.4. Untargeted Metabolomic Differences Between NJ and DLY Longissimus Dorsi Muscles

Untargeted metabolomic analysis was performed to characterize small-molecule differences between NJ and DLY longissimus dorsi muscles. Quality-control assessment showed tight clustering of pooled QC samples in PCA. The mean QC CVs of the retained metabolic features were 9.84% and 10.20% in negative- and positive-ion modes, respectively; 87.9% and 84.9% of the retained features had QC CVs ≤ 20%, respectively, and all retained features had QC CVs < 30%. Pairwise Pearson correlation coefficients among pooled QC samples were 0.994 in both ionization modes. Together, these results indicated stable analytical performance of the untargeted metabolomic dataset (Figure S2).

PCA of the biological samples showed an apparent tendency toward NJ–DLY separation in negative-ion mode, whereas only partial separation with substantial overlap was observed in positive-ion mode. PC1 and PC2 explained 26.70% and 19.76% of the total variance in negative-ion mode and 27.01% and 17.41% in positive-ion mode, respectively (Figure S3). Thus, the unsupervised PCA descriptively suggested differences in the overall sample distribution between NJ and DLY, although the extent of separation differed between ionization modes.

After quality-control filtering, 678 and 1179 ion-mode-specific metabolic features were retained for differential-abundance analysis in negative- and positive-ion modes, respectively. Benjamini–Hochberg correction was applied jointly across all 1857 ion-mode-specific features. Using the predefined criteria of BH-adjusted *q* ≤ 0.05 and FC ≥ 1.5 or FC ≤ 1/1.5, 107 features met the differential-abundance criteria in negative-ion mode, including 18 with higher and 89 with lower abundance in NJ. In positive-ion mode, 172 features met these criteria, including 66 with higher and 106 with lower abundance in NJ. Thus, a total of 279 features constituted the primary FDR- and fold-change-supported differential-abundance set. Complete differential-abundance statistics are provided in Table S5, and the 279 features meeting these criteria are listed in Table S6.

Exploratory PLS-DA showed visual separation between NJ and DLY in the supervised score plots for both ionization modes, with R2Y/Q2Y values of 0.99/0.86 for the negative-ion dataset and 0.99/0.81 for the positive-ion dataset. However, the corresponding 200-permutation tests yielded R2 intercepts of 0.89 and 0.93 and Q2 intercepts of −0.85 and −0.76 in negative- and positive-ion modes, respectively (Figure S4). The high R2 intercepts indicated susceptibility of the supervised models to overfitting. Accordingly, PLS-DA was treated as an exploratory supervised analysis, whereas differential metabolic features were defined using the FDR-controlled criteria described above. VIP scores were reported as supplementary multivariate descriptors. Among the 279 FDR- and fold-change-supported features, 269 additionally had VIP > 1, and this supplementary subset is provided in Table S7.

**Figure 4:**
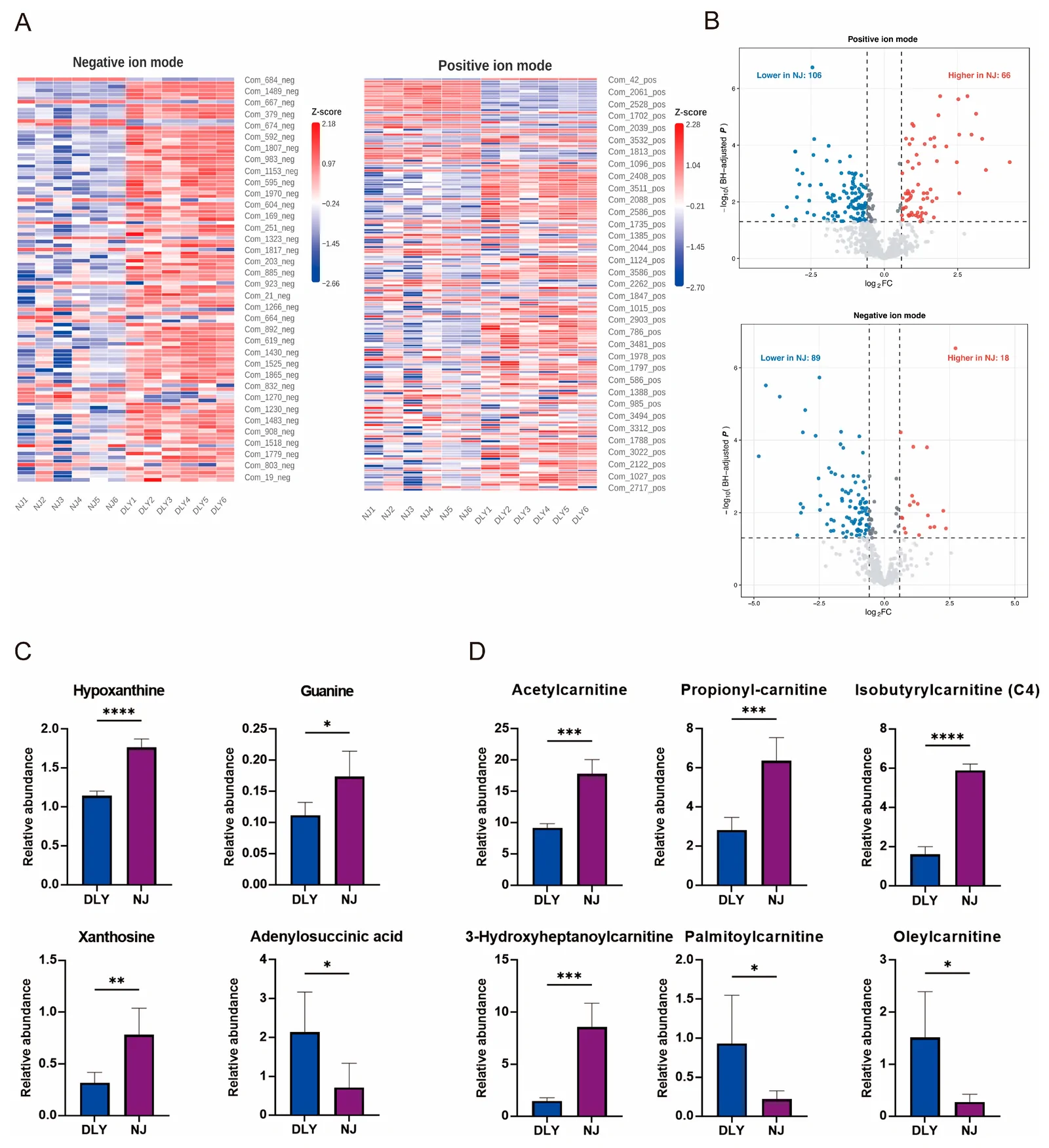
Untargeted metabolomic profiling and representative metabolic features differing between NJ and DLY longissimus dorsi muscles. (**A**) Hierarchical clustering heatmaps of metabolic features meeting the differential-abundance criteria in negative- and positive-ion modes. Relative abundances are displayed as row-wise Z-scores. (**B**) Volcano plots of metabolic features in negative- and positive-ion modes. The *x*-axis represents log_2_ fold change (NJ/DLY), and the y-axis represents −log_10_(BH-adjusted *q* value). Differentially abundant features were defined by BH-adjusted *q* ≤ 0.05 and FC ≥ 1.5 or FC ≤ 1/1.5. (**C**) Relative abundances of representative purine-related metabolic features annotated as hypoxanthine, xanthosine, guanine, and adenylosuccinic acid. (**D**) Relative abundances of representative acylcarnitine-related features annotated as acetylcarnitine, propionyl-carnitine, isobutyrylcarnitine (C4), 3-hydroxyheptanoylcarnitine, palmitoylcarnitine, and oleylcarnitine. Values in panels C and D are presented as mean ± SD (*n* = 6 biological replicates per group). Statistical significance was based on BH-adjusted *q* values: * *q* < 0.05, ** *q* < 0.01, *** *q* < 0.001, and **** *q* < 0.0001. Individual biological observations and detailed statistical and identification information for the representative metabolic features are provided in Table S8. NJ, New Jilin Black pigs; DLY, Duroc × Landrace × Yorkshire pigs; FC, fold change; BH, Benjamini–Hochberg.

Hierarchical clustering heatmaps were used to visualize the relative abundance patterns of the 279 FDR- and fold-change-supported features across individual NJ and DLY samples in negative- and positive-ion modes (Figure 4A), whereas volcano plots summarized their distributions according to fold change and BH-adjusted significance (Figure 4B). The heatmaps were used descriptively to visualize relative abundance patterns within the preselected FDR- and fold-change-supported feature set.

Metabolite names reported below denote untargeted annotations of metabolic features and do not represent authentic-standard-confirmed identifications.

Among the FDR- and fold-change-supported metabolic features, four representative purine-related features were highlighted in relation to the targeted IMP phenotype. A feature annotated as hypoxanthine showed higher relative abundance in NJ than in DLY (log_2_FC = 0.625, 95% CI: 0.474–0.776, raw *p* = 6.65 × 10^−7^, BH-adjusted *q* = 6.10 × 10^−5^). Features annotated as xanthosine (log_2_FC = 1.276, 95% CI: 0.696–1.857, raw *p* = 3.93 × 10^−4^, *q* = 0.00571) and guanine (log_2_FC = 0.619, 95% CI: 0.200–1.038, raw *p* = 0.00726, *q* = 0.0429) also showed higher relative abundance in NJ. In contrast, a feature annotated as adenylosuccinic acid showed lower relative abundance in NJ (log_2_FC = −1.951, 95% CI: −3.185 to −0.717, raw *p* = 0.00467, *q* = 0.0314) (Figure 4C). Collectively, these representative features showed a heterogeneous purine-related pattern, with higher relative abundance of the hypoxanthine-, xanthosine-, and guanine-related features but lower relative abundance of the adenylosuccinic acid-related feature in NJ.

Several representative acylcarnitine-related features within the FDR- and fold-change-supported differential set also differed between NJ and DLY muscles. Features annotated as acetylcarnitine (log_2_FC = 0.951, 95% CI: 0.759–1.144, raw *p* = 1.15 × 10^−7^, *q* = 1.79 × 10^−5^), propionyl-carnitine (log_2_FC = 1.181, 95% CI: 0.814–1.548, raw *p* = 1.19 × 10^−5^, *q* = 4.51 × 10^−4^), isobutyrylcarnitine (C4) (log_2_FC = 1.902, 95% CI: 1.618–2.186, raw *p* = 3.41 × 10^−9^, *q* = 1.86 × 10^−6^), and 3-hydroxyheptanoylcarnitine (log_2_FC = 2.521, 95% CI: 2.117–2.926, raw *p* = 7.73 × 10^−9^, *q* = 2.39 × 10^−6^) showed higher relative abundance in NJ. In contrast, features annotated as palmitoylcarnitine (log_2_FC = −1.910, 95% CI: −3.066 to −0.753, raw *p* = 0.00355, *q* = 0.0262) and oleylcarnitine (log_2_FC = −2.560, 95% CI: −3.829 to −1.291, raw *p* = 8.23 × 10^−4^, *q* = 0.00910) showed lower relative abundance in NJ (Figure 4D). Thus, the representative acylcarnitine-related features displayed a heterogeneous directional pattern, with the acetyl-, propionyl-, isobutyryl-, and 3-hydroxyheptanoylcarnitine-related features higher in NJ, whereas the long-chain palmitoyl- and oleylcarnitine-related features were lower.

Five additional representative features from this differential set, annotated as amino acid derivatives or small peptide-like metabolites, are presented in Figure S5. Features annotated as L-beta-homoleucine and N-lauroyl glutamic acid showed higher relative abundance in NJ, whereas features annotated as N-acetylmethionine, glutamylthreonine, and methionyl-glycine showed lower relative abundance in NJ. Thus, these features also exhibited mixed directional changes rather than a uniform increase or decrease in NJ. Given their untargeted annotation, these features were presented descriptively as supplementary indicators of broader metabolic differences and were not considered direct measurements of free amino acid concentrations or confirmed taste-active peptides. Statistical results, individual biological observations, and available identification information for these five features, together with the ten representative features shown in Figure 4C,D, are provided in Table S8.

### 3.5. Cross-Omics Comparison of Purine-Related Molecular Features in NJ and DLY Longissimus Dorsi Muscles

**Figure 5:**
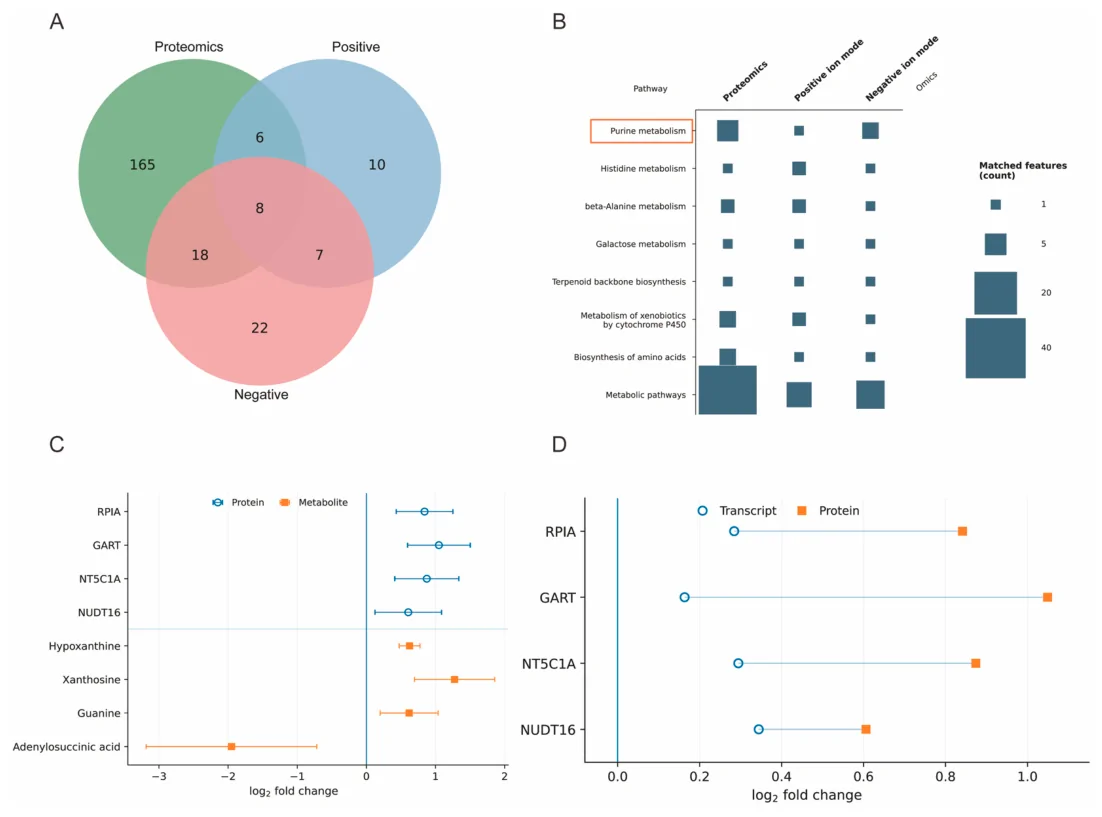
Cross-omics comparison of purine-related molecular features in the longissimus dorsi muscle of NJ and DLY pigs. (**A**) Venn diagram showing shared KEGG pathway annotations among the nominal exploratory protein set and the FDR-supported differential metabolite feature sets detected in positive- and negative-ion modes. Eight KEGG pathway annotations were represented in all three feature sets. (**B**) Matrix visualization showing the numbers of molecular features from the proteomic, positive-ion metabolomic, and negative-ion metabolomic datasets mapped to the eight shared KEGG pathway annotations. Square size represents the number of matched molecular features in each dataset. (**C**) Group-level NJ/DLY log_2_FC estimates and 95% confidence intervals for four exploratory purine-related candidate proteins (RPIA, GART, NT5C1A, and NUDT16) and four representative purine-related metabolite features annotated as hypoxanthine, xanthosine, guanine, and adenylosuccinic acid. None of the four candidate proteins met the proteome-wide BH-adjusted *q* ≤ 0.05 criterion, whereas all four metabolite features met the predefined FDR and fold-change criteria. Panel C presents group-level effect estimates; individual-level protein–IMP or protein–metabolite associations were not estimated. (**D**) Descriptive comparison of transcriptomic and proteomic NJ/DLY log_2_FC values for RPIA, GART, NT5C1A, and NUDT16. Lines connect transcript- and protein-level estimates for the same candidate for visual comparison and do not denote statistical associations. None of the four corresponding transcripts met the predefined transcriptomic significance criterion after multiple-testing correction. IMP, inosine 5′-monophosphate; KEGG, Kyoto Encyclopedia of Genes and Genomes; NJ, New Jilin Black pigs; DLY, Duroc × Landrace × Yorkshire pigs; FC, fold change; BH, Benjamini–Hochberg; FDR, false discovery rate.

Cross-omics comparison was performed to examine shared functional annotations and group-level patterns across the proteomic and metabolomic datasets. KEGG pathway annotations of the nominal exploratory protein set (*n* = 250) and the FDR-supported differential metabolite feature sets detected in negative-ion (*n* = 107) and positive-ion (*n* = 172) modes were compared. Eight KEGG pathway annotations were represented in all three feature sets, including purine metabolism, histidine metabolism, beta-alanine metabolism, galactose metabolism, terpenoid backbone biosynthesis, metabolism of xenobiotics by cytochrome P450, biosynthesis of amino acids, and metabolic pathways (Figure 5A). The numbers of molecular features from each dataset mapped to these shared annotations are summarized in Figure 5B. Because IMP was the targeted purine-nucleotide phenotype of this study, purine metabolism was subsequently examined as a phenotype-relevant functional context.

Within this purine-related context, the exploratory candidate proteins RPIA, GART, NT5C1A, and NUDT16 all showed nominally higher abundance in NJ than in DLY, although none met the proteome-wide BH-adjusted *q* ≤ 0.05 criterion, as described in Section 3.3. In contrast, the metabolite features annotated as hypoxanthine, xanthosine, guanine, and adenylosuccinic acid all met the predefined FDR and fold-change criteria; the hypoxanthine-, xanthosine-, and guanine-related features showed higher relative abundance in NJ, whereas the adenylosuccinic acid-related feature showed lower relative abundance, as described in Section 3.4. The corresponding NJ/DLY log_2_FC estimates and 95% confidence intervals for these protein and metabolite features are presented together in Figure 5C. Figure 5C summarizes the NJ/DLY group-level effect estimates and their 95% confidence intervals across the proteomic and metabolomic layers. Together, these results show parallel between-group differences in several purine-related molecular features across the two analytical layers.

Transcript-level results corresponding to the four exploratory purine-related protein candidates were further examined. RPIA (log_2_FC = 0.284, adjusted *p* = 0.519), GART (log_2_FC = 0.163, adjusted *p* = 0.916), NT5C1A (log_2_FC = 0.294, adjusted *p* = 0.845), and NUDT16 (log_2_FC = 0.344, adjusted *p* = 0.783) all showed positive NJ/DLY transcript-level log_2_FC values, consistent in direction with their corresponding nominal protein abundance differences (Figure 5D). However, none met the predefined transcriptomic significance criterion after multiple-testing correction. These transcript-level results were therefore treated as descriptive directional information. Complete gene-level differential-expression results are provided in Table S3.

Overall, the cross-omics comparison identified parallel group-level differences in several purine-related molecular features accompanying the higher measured IMP content in NJ muscle, providing an exploratory and hypothesis-generating context for subsequent interpretation.

### 3.6. Proposed Exploratory Molecular Framework Accompanying Higher IMP Content in NJ Longissimus Dorsi Muscle

**Figure 6:**
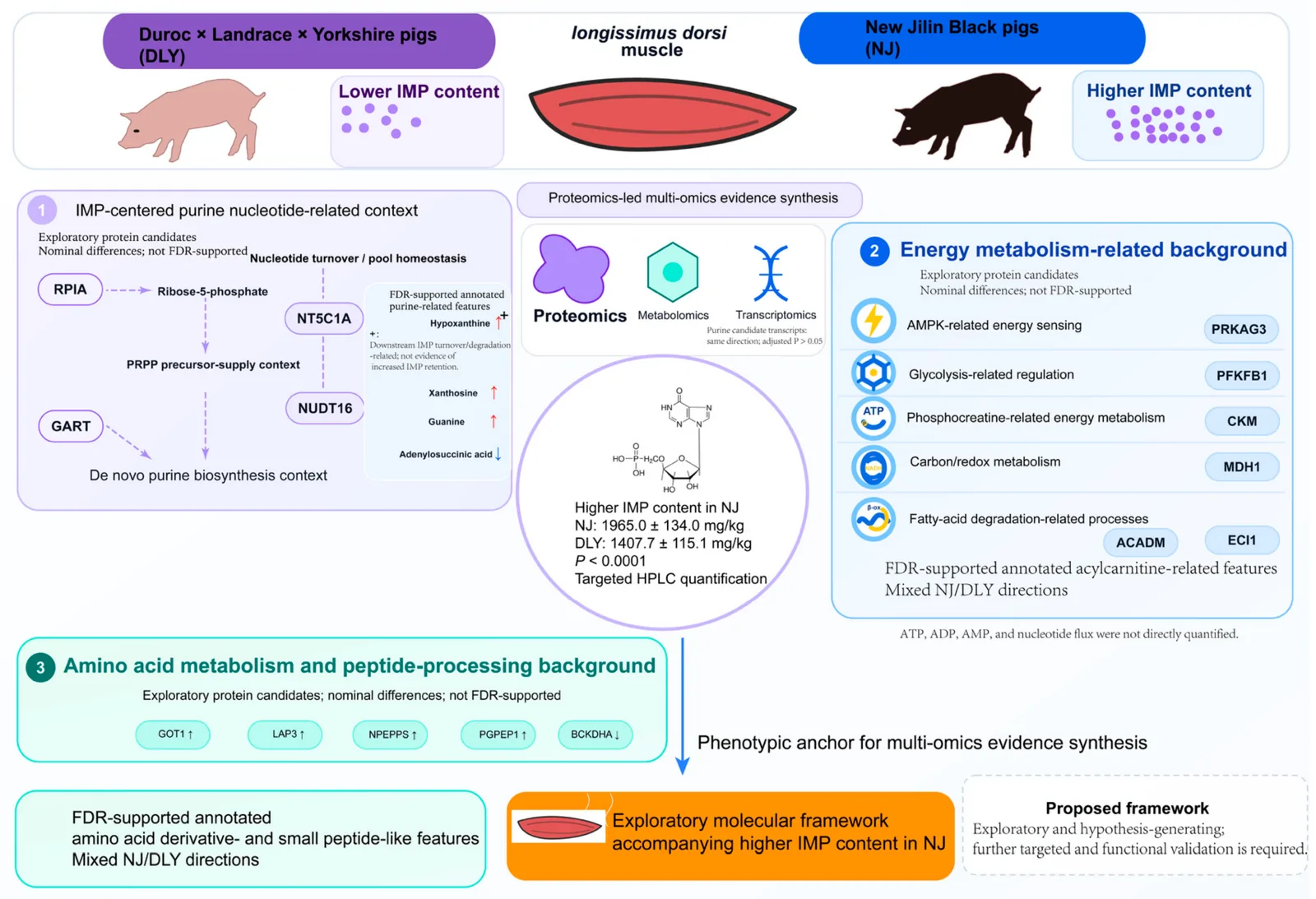
Proposed exploratory molecular framework accompanying higher IMP content in the longissimus dorsi muscle of New Jilin Black pigs. Higher targeted IMP content in NJ muscle was used as the phenotypic anchor for proteomics-led multi-omics evidence synthesis. The molecular features were organized into three functional contexts. (1) The IMP-centered purine nucleotide-related context included the exploratory protein candidates RPIA, GART, NT5C1A, and NUDT16, none of which met the proteome-wide FDR criterion, together with FDR-supported untargeted features annotated as hypoxanthine, xanthosine, guanine, and adenylosuccinic acid. RPIA was considered in relation to the ribose-5-phosphate/PRPP precursor-supply context, GART to de novo purine biosynthesis, and NT5C1A and NUDT16 to nucleotide turnover or nucleotide-pool homeostasis. The hypoxanthine-related feature was considered in the context of downstream IMP turnover/degradation and was not interpreted as evidence of increased IMP retention. The corresponding transcripts of the four purine-related protein candidates showed the same NJ/DLY direction as the nominal protein abundance differences but were not statistically significant after multiple-testing correction. (2) The energy metabolism-related context included the exploratory protein candidates PRKAG3, PFKFB1, CKM, MDH1, ACADM, and ECI1, none of which met the proteome-wide FDR criterion, together with FDR-supported annotated acylcarnitine-related features showing mixed NJ/DLY directions. ATP, ADP, AMP, and nucleotide flux were not directly quantified. (3) The amino acid metabolism- and peptide-processing-related context included the exploratory protein candidates GOT1, LAP3, NPEPPS, PGPEP1, and BCKDHA, none of which met the proteome-wide FDR criterion, together with FDR-supported untargeted features annotated as amino acid derivatives or small peptide-like metabolites showing mixed NJ/DLY directions. Free amino acids and confirmed taste-active peptides were not directly measured. Dashed arrows indicate annotation-based or established biochemical contexts rather than measured molecular interactions, metabolic flux, or causal effects. The framework is phenotype-oriented, exploratory, and hypothesis-generating and does not represent a validated regulatory pathway or causal mechanism. IMP, inosine 5′-monophosphate; PRPP, 5-phosphoribosyl-1-pyrophosphate; NJ, New Jilin Black pigs; DLY, Duroc × Landrace × Yorkshire pigs; FDR, false discovery rate.

Based on the targeted IMP phenotype, the exploratory proteomic results, FDR-supported metabolomic differences, and complementary transcript-level information, an exploratory molecular framework was constructed to summarize molecular features accompanying the higher measured IMP content in NJ longissimus dorsi muscle (Figure 6). The higher targeted IMP content was used as the phenotypic anchor, and the selected molecular features were organized into three molecular contexts: an IMP-centered purine nucleotide-related context, an energy metabolism-related context, and an amino acid metabolism- and peptide-processing-related context.

Within the IMP-centered purine nucleotide-related context, RPIA, GART, NT5C1A, and NUDT16 were retained as exploratory protein candidates. All four showed nominally higher abundance in NJ but did not meet the proteome-wide BH-adjusted *q* ≤ 0.05 criterion. Based on their functional annotations, RPIA was considered in relation to the ribose-5-phosphate/PRPP precursor-supply context and GART to de novo purine biosynthesis [28,29], whereas NT5C1A and NUDT16 were considered in relation to nucleotide hydrolysis or nucleotide-pool homeostasis [31,32,33]. At the metabolite level, FDR-supported features annotated as hypoxanthine, xanthosine, and guanine showed higher relative abundance in NJ, whereas the feature annotated as adenylosuccinic acid showed lower relative abundance. The corresponding transcripts of RPIA, GART, NT5C1A, and NUDT16 showed the same NJ/DLY direction as the nominal protein abundance differences but were not statistically significant after multiple-testing correction. The higher hypoxanthine-related feature is also consistent with downstream IMP turnover/degradation and therefore does not distinguish increased IMP retention from ongoing IMP degradation. Together, these observations indicate group-level differences in purine-related molecular features, without resolving the direction of nucleotide flux.

The energy metabolism-related context included the exploratory protein candidates PRKAG3, CKM, PFKFB1, MDH1, ACADM, and ECI1 together with several FDR-supported acylcarnitine-related metabolic features. Each of these protein candidates showed nominally higher abundance in NJ but did not meet the proteome-wide FDR criterion. Their functional annotations encompassed energy sensing, ATP–phosphocreatine buffering, glycolytic regulation, carbon and redox metabolism, and fatty-acid degradation-related processes. The accompanying acylcarnitine-related features showed mixed directional differences between NJ and DLY: the acetylcarnitine-, propionyl-carnitine-, isobutyrylcarnitine-, and 3-hydroxyheptanoylcarnitine-related features showed higher relative abundance in NJ, whereas the palmitoylcarnitine- and oleylcarnitine-related features showed lower relative abundance. Together, these features characterized an energy metabolism-related molecular context accompanying the IMP phenotype.

The amino acid metabolism- and peptide-processing-related context included the exploratory protein candidates GOT1, LAP3, NPEPPS, PGPEP1, and BCKDHA together with FDR-supported untargeted features annotated as amino acid derivatives or small peptide-like metabolites. GOT1, LAP3, NPEPPS, and PGPEP1 showed nominally higher abundance in NJ, whereas BCKDHA showed nominally lower abundance; none met the proteome-wide FDR criterion. The annotated metabolic features likewise exhibited mixed directional differences between groups. Together, these features characterized a broader amino acid metabolism- and peptide-processing-related molecular context. Free amino acids and confirmed taste-active peptides were not measured.

Overall, the proposed framework integrates the targeted IMP phenotype with exploratory protein candidates, FDR-supported annotated metabolite features, and complementary transcript-level information. It provides a hypothesis-generating summary of molecular features accompanying the higher measured IMP content in NJ muscle.

## 4. Discussion

This study showed that IMP content was significantly higher in the longissimus dorsi muscle of NJ pigs than in DLY pigs. IMP is an important non-volatile umami-associated nucleotide in meat and contributes to meat taste together with other taste-active compounds [12,34]. Importantly, IMP can act synergistically with free glutamate in umami perception [16]. In the present study, free amino acids, including glutamate and aspartate, were not quantified; therefore, the potential IMP–glutamate synergy could not be evaluated, and EUC could not be calculated [35]. Electronic tongue analysis and sensory evaluation were also not performed; these approaches have been used to characterize umami-related sensory properties in meat [36]. Accordingly, the present findings demonstrate higher IMP content in NJ muscle but do not establish stronger sensory umami intensity or superior sensory quality. In this manuscript, the term “IMP-related biochemical umami potential” is used only as a restricted biochemical descriptor of the higher level of this umami-associated nucleotide, rather than as a proxy for actual sensory umami intensity.

Using IMP content as the core phenotypic anchor, this study applied a proteomics-led multi-omics strategy to characterize molecular features accompanying the higher IMP content observed in NJ muscle. Proteomics was used as the primary discovery layer because it directly profiles protein abundance and can provide a protein-level view of molecular components associated with metabolic processes, complementing transcript- and metabolite-level measurements [23,37,38]. Following proteome-wide multiple-testing correction, only PTER and EML2 met both the BH-adjusted *q* ≤ 0.05 and fold-change criteria. Neither PTER nor EML2 was prioritized in the IMP-centered exploratory framework because the functional annotations considered in the present analysis did not provide a clear basis for linking either protein to the IMP-centered purine nucleotide context. Thus, PTER and EML2 represent the only FDR-supported protein-abundance differences detected between NJ and DLY in the present dataset, but their relevance to the higher IMP phenotype remains unclear. The broader set of 250 proteins meeting the nominal screening criteria was retained for exploratory functional annotation and candidate prioritization. Within this broader set, RPIA, GART, NT5C1A, and NUDT16 were treated as exploratory candidate molecular features rather than FDR-supported differentially abundant proteins. Because their prioritization was phenotype-oriented and post hoc, these candidates were used for hypothesis-generating biological interpretation rather than as an independent statistical test of association with IMP. Metabolomics was used to characterize accompanying small-molecule differences, whereas transcriptomics provided complementary directional information for the selected candidates; notably, the corresponding transcripts of RPIA, GART, NT5C1A, and NUDT16 changed in the same NJ/DLY direction as their protein-level abundance differences but did not reach the predefined transcriptomic significance criterion. Thus, the proteomics-led multi-omics evidence synthesis provides an exploratory molecular context accompanying the higher IMP phenotype rather than evidence of a causal regulatory mechanism.

Given that IMP is a purine nucleotide, the four FDR-supported purine-related metabolite features provided a complementary small-molecule context for interpreting the targeted IMP phenotype. Features annotated as hypoxanthine, xanthosine, and guanine showed higher relative abundance in NJ, whereas the feature annotated as adenylosuccinic acid showed lower relative abundance. Because these assignments were obtained from untargeted metabolomic annotation without confirmation against authentic standards, they are interpreted here as annotated purine-related features rather than definitively identified metabolites. The higher xanthosine- and guanine-related features indicate that the between-group differences extended beyond IMP and its immediate degradation products to a broader purine metabolic context involving guanine-related metabolites; however, these abundance differences alone do not indicate increased metabolic flux toward guanine nucleotides. More importantly, the higher hypoxanthine-related feature should not be interpreted solely as support for the higher IMP phenotype. Hypoxanthine is a downstream product of IMP degradation through inosine [39], and its higher abundance is therefore also compatible with greater downstream IMP degradation. Nevertheless, higher IMP levels and progressive accumulation of inosine and hypoxanthine are not necessarily mutually exclusive in postmortem muscle; a previous time-course study showed that IMP increased rapidly and remained relatively high while inosine and hypoxanthine progressively accumulated [40]. Thus, the concurrent higher measured IMP content and higher hypoxanthine-related feature in NJ are compatible with relatively high IMP content coexisting with ongoing downstream degradation, but they do not establish whether IMP formation, retention, or degradation occurred at a higher rate in NJ. The feature annotated as adenylosuccinic acid (adenylosuccinate) lies within the IMP-to-AMP branch of purine nucleotide metabolism [29], in which adenylosuccinate is an intermediate between IMP and AMP. Its lower abundance in NJ could reflect reduced formation from IMP, greater downstream utilization toward AMP, or other determinants of the adenylosuccinate pool. Therefore, this static abundance difference cannot determine whether flux through the IMP-to-AMP branch was increased or decreased. Because postmortem ATP degradation and subsequent nucleotide turnover are time- and temperature-dependent, the interval from slaughter to freezing and subsequent storage conditions can influence the measured levels of IMP and its downstream degradation products [34,39,41]. Accordingly, comparable postmortem handling between NJ and DLY was important for minimizing processing-related variation. Although all animals were slaughtered on the same day and within the same slaughter batch, samples were collected within a standardized early-postmortem window (within 40 min), immediately snap-frozen, and subsequently handled under standardized conditions for each analysis; serial postmortem sampling was not performed. Consequently, the kinetics of IMP formation and degradation could not be resolved. Hypoxanthine has also been associated with bitter aftertaste in comparative meat studies [15]; however, no sensory consequence can be inferred from the present results because neither sensory evaluation nor taste-activity assessment was performed. Collectively, these findings show NJ–DLY differences in the relative-abundance pattern of several annotated purine-related features accompanying the IMP phenotype, while the underlying direction and kinetics of nucleotide flux remain unresolved.

Among the exploratory purine-related protein candidates, RPIA and GART provide potential biochemical contexts related to nucleotide precursor supply and de novo purine biosynthesis, respectively. RPIA showed nominally higher abundance in NJ but did not meet the proteome-wide FDR criterion; moreover, it was not directly annotated to purine metabolism in KEGG. Instead, RPIA was associated with the pentose phosphate pathway and ribose-5-phosphate isomerase activity. Ribose-5-phosphate provides a precursor context for the formation of PRPP, which is an essential substrate for de novo purine nucleotide biosynthesis [28,29]. Thus, the RPIA difference is most appropriately interpreted as an exploratory indication of a potentially different ribose-5-phosphate/PRPP precursor-supply context rather than evidence of increased purine synthesis. GART, which also showed nominally higher abundance without reaching the proteome-wide FDR threshold, participates directly in early reactions of de novo purine biosynthesis upstream of IMP [29]. Its abundance difference therefore provides a plausible de novo purine-related molecular context for the NJ–DLY comparison, but protein abundance alone does not establish increased GART activity or greater de novo purine flux. This distinction is particularly important because postmortem muscle IMP is closely linked to ATP degradation and AMP-related nucleotide metabolism during the conversion of muscle to meat [18]. Accordingly, the observed RPIA and GART abundance differences cannot establish that enhanced de novo purine synthesis was responsible for the higher measured IMP content in NJ. Direct measurements of PRPP and other pathway intermediates, enzyme activities, or metabolic flux would be required to test this hypothesis.

NT5C1A and NUDT16 were also retained as exploratory candidates because both are related to nucleotide hydrolysis or nucleotide-pool homeostasis, although neither met the proteome-wide FDR threshold. NT5C1A is a cytosolic 5′-nucleotidase that can hydrolyze AMP to adenosine and is involved in adenine nucleotide metabolism in skeletal muscle [31]. Its nominally higher abundance in NJ therefore provides an exploratory protein-level context for differences in nucleotide turnover. Importantly, however, this observation should not be interpreted as evidence that NT5C1A contributed to higher IMP retention. Likewise, the higher hypoxanthine-related feature should not be directly attributed to the nominally higher NT5C1A abundance, because the present data do not establish a direct relationship between these two observations. At the same time, protein abundance alone does not establish NT5C1A enzymatic activity or substrate-specific flux, and inosine was not quantitatively characterized as part of a targeted IMP-degradation series. NUDT16 has been characterized as a (deoxy)inosine diphosphatase, while structural and biochemical studies indicate that its activity can be regulated by IMP [32,33]. These findings do not establish NUDT16 as an enzyme directly converting IMP along the IMP–inosine–hypoxanthine degradation sequence. Thus, its nominally higher abundance may indicate a difference in nucleotide-pool homeostasis, but it does not demonstrate direct conversion or degradation of IMP. Accordingly, the NT5C1A and NUDT16 abundance differences are best interpreted as exploratory protein-level features relevant to nucleotide hydrolysis and pool regulation, rather than as evidence that either protein promoted IMP accumulation or that downstream IMP degradation was increased in NJ.

Energy metabolism-related molecular features were examined as a complementary context because postmortem IMP formation and turnover occur within the broader process of ATP degradation and AMP-related nucleotide metabolism during the conversion of muscle to meat [30,42,43]. PRKAG3, CKM, PFKFB1, MDH1, ACADM, and ECI1 each showed nominally higher abundance in NJ, but none met the proteome-wide FDR threshold. Based on their functional annotations and established biochemical roles, these proteins are related to several aspects of energy metabolism, including AMPK-related energy sensing, ATP–phosphocreatine buffering, glycolytic regulation, carbon and oxidation–reduction metabolism, and fatty-acid degradation-related processes [43,44,45,46,47,48]. These abundance differences therefore represent exploratory protein-level differences in the energy-metabolism-related molecular context between NJ and DLY. However, they do not demonstrate activation or increased flux of the corresponding pathways; for example, higher PRKAG3 abundance does not by itself establish greater AMPK signaling activity, and abundance differences in CKM, PFKFB1, MDH1, ACADM, or ECI1 do not establish corresponding changes in enzyme activity or pathway flux. At the metabolite level, several features annotated as acylcarnitines also differed between NJ and DLY. Acylcarnitines are associated with fatty-acid transport and mitochondrial lipid metabolism [49,50], but differences in their steady-state abundance alone cannot establish increased or decreased β-oxidation flux. Moreover, because ATP, ADP, AMP, and related energy intermediates were not quantified as a targeted series and enzyme activities or metabolic flux were not measured, the combined protein and metabolite patterns cannot demonstrate enhanced ATP-to-AMP-to-IMP conversion or a causal contribution of altered energy metabolism to the higher measured IMP content in NJ. Accordingly, this context is interpreted as a complementary energy-metabolism-related molecular context accompanying the NJ–DLY difference in IMP content.

The amino acid metabolism- and peptide-processing-related features were examined as a secondary molecular context. Free amino acids and some peptides can contribute to the non-volatile taste characteristics of meat [12]. However, free amino acids were not quantified, and taste-active peptides were neither specifically identified nor functionally evaluated in the present study. At the protein level, GOT1, LAP3, NPEPPS, and PGPEP1 showed nominally higher abundance in NJ, whereas BCKDHA showed nominally lower abundance; none met the proteome-wide FDR threshold. Based on the functional annotations in the present dataset, GOT1 was associated with amino acid-related pathways, including alanine, aspartate and glutamate metabolism, whereas LAP3, NPEPPS, and PGPEP1 were associated with peptidase, proteolysis, or peptide-processing functions, and BCKDHA with branched-chain amino acid degradation. These abundance differences indicate exploratory protein-level differences in the amino acid metabolism- and peptide-processing-related context without establishing increased amino acid release, peptide hydrolysis, or metabolic flux. At the metabolite level, five FDR-supported untargeted features annotated as L-beta-homoleucine, N-acetylmethionine, glutamylthreonine, methionyl-glycine, and N-lauroyl glutamic acid also differed between NJ and DLY. Although these five features were FDR-supported statistically, their chemical assignments were based on untargeted metabolomic annotation and were not confirmed against authentic standards. Accordingly, these features should not be considered direct measurements of free amino acid concentrations or confirmed taste-active peptides. In particular, free glutamate and aspartate were not quantified; therefore, IMP–glutamate synergy could not be assessed [16]. Overall, these findings provide a complementary molecular context reflecting broader NJ–DLY differences in amino acid metabolism- and peptide-processing-related features, without direct evidence regarding taste-active compound production.

The transcriptomic layer provided complementary directional information for the four purine-related protein candidates. The corresponding transcripts of RPIA, GART, NT5C1A, and NUDT16 all showed positive NJ/DLY log_2_FC values, consistent in direction with their respective nominal protein abundance differences. However, none of the four transcripts met the predefined transcriptomic significance criterion after multiple-testing correction. Thus, the transcript-level results were interpreted as descriptive directional information rather than statistically significant confirmation of the protein-level findings. Accordingly, RPIA, GART, NT5C1A, and NUDT16 remain exploratory protein candidates based on their nominal protein-abundance differences and functional annotations.

Cross-omics comparison integrated evidence of different statistical and evidentiary strengths across analytical layers. Targeted HPLC quantification directly established the higher IMP phenotype in NJ, whereas RPIA, GART, NT5C1A, and NUDT16 remained phenotype-oriented exploratory protein candidates that met the nominal screening criteria but did not pass proteome-wide FDR control. In contrast, the representative purine-related metabolite features met the FDR-based statistical criteria in the untargeted metabolomic analysis, although their chemical assignments remained based on untargeted annotation rather than confirmation with authentic standards. The corresponding transcripts showed the same NJ/DLY direction as the protein abundance differences but did not meet the predefined transcriptomic significance criterion. Accordingly, the cross-omics comparison focused on NJ/DLY group-level effect estimates and their 95% confidence intervals rather than individual-level protein–IMP or protein–metabolite associations. The resulting group-level patterns indicate that several purine-related molecular features differed between NJ and DLY across analytical layers without resolving direct molecular interactions, coordinated regulation, or metabolic flux. Moreover, although purine metabolism was among the shared KEGG functional contexts identified across the exploratory protein set and the FDR-supported metabolite feature sets, it was examined further because IMP itself was the targeted purine-nucleotide phenotype. Thus, the further examination of purine metabolism represented phenotype-oriented biological prioritization centered on the targeted IMP phenotype. Taken together, the integrated findings provide a hypothesis-generating molecular context accompanying the higher measured IMP content in NJ.

Several limitations should be considered when interpreting these findings. First, the animals were selected at a similar target body weight rather than being age-matched. Consequently, NJ pigs were older at slaughter than DLY pigs, and breed and age were not independently balanced in the present design. Because slaughter age has been associated with variation in porcine longissimus muscle characteristics and meat-quality-related biochemical traits [51,52], age-related contributions to the observed IMP and omics differences cannot be excluded. The possible contribution of age therefore cannot be separated from the breed-associated differences in the present study, and the findings should be interpreted within this similar-body-weight comparative design rather than as evidence of breed effects independent of age. Second, the proteomic and transcriptomic analyses included only four biological replicates per group. This limited sample size reduces statistical power and precision and increases the uncertainty associated with exploratory molecular signals; notably, the IMP-centered candidate proteins did not meet the proteome-wide FDR threshold, and their corresponding transcripts were not statistically significant after multiple-testing correction. Third, the purine-related, acylcarnitine-related, amino acid derivative-, and small peptide-like metabolic features discussed here were derived from untargeted metabolomic annotation and were not confirmed against authentic standards; their chemical assignments should therefore remain provisional. Fourth, although postmortem handling and storage conditions were standardized across the two groups, samples were collected within a single standardized early-postmortem window rather than through serial postmortem sampling, and ATP, ADP, AMP, PRPP, inosine, and other relevant nucleotide intermediates were not quantified as a targeted series. Enzyme activities and metabolic flux were also not measured. Accordingly, the present data cannot resolve the relative contributions or temporal kinetics of IMP formation, retention, and degradation. Fifth, free glutamate, free aspartate, other free taste-active amino acids, and taste-active peptides were not quantified, and EUC, taste activity values, electronic-tongue analysis, and sensory evaluation were not performed. The higher IMP content observed in NJ therefore cannot be extrapolated to stronger sensory umami or superior sensory quality. In addition, because only intact male pigs were included, the generalizability of these findings to females or castrated males remains to be determined.

Future studies should test the present hypothesis-generating framework using larger, independently sampled populations and experimental designs that better separate breed effects from age-related effects. Targeted postmortem time-course profiling of ATP, ADP, AMP, IMP, inosine, hypoxanthine, PRPP, adenylosuccinic acid, and other relevant purine intermediates, together with measurements of key enzyme activities and, where feasible, metabolic flux, would help distinguish the relative contributions of IMP formation, retention, and degradation. Authentic-standard confirmation and targeted quantification of key purine metabolites would further strengthen the chemical evidence underlying the purine-related interpretation. Functional studies of RPIA, GART, NT5C1A, and NUDT16 are also required to determine whether their abundance differences have biological consequences for nucleotide metabolism. In parallel, targeted quantification of free glutamate, aspartate, other taste-active free amino acids, and taste-active peptides, together with EUC and taste-activity assessment, electronic-tongue analysis, and sensory evaluation, would help determine whether the higher IMP content translates into differences in perceived umami. Independent replication of the exploratory protein candidates and cross-omics patterns will also be important given the limited proteomic and transcriptomic sample sizes in the present study. Overall, the present study provides a prioritized set of candidate molecular features and metabolic contexts accompanying the higher measured IMP content in NJ muscle, while the mechanisms underlying this phenotype and its sensory significance remain to be established experimentally.

## 5. Conclusions

In the present similar-body-weight comparison, targeted HPLC analysis showed that IMP content was higher in the longissimus dorsi muscle of NJ pigs than in DLY pigs. Proteomics-led multi-omics analysis further characterized a set of molecular features accompanying this phenotype. RPIA, GART, NT5C1A, and NUDT16 were retained as exploratory purine-related protein candidates based on their nominal abundance differences and functional annotations, but none met the proteome-wide FDR threshold. Untargeted metabolomics identified FDR-supported differences in several annotated purine-related features; features annotated as hypoxanthine, xanthosine, and guanine showed higher relative abundance in NJ, whereas the feature annotated as adenylosuccinic acid showed lower relative abundance. These metabolite patterns indicate a broader difference in purine-related metabolic context but do not resolve whether IMP formation, retention, or degradation differed between groups. The corresponding transcripts of the four protein candidates showed the same NJ/DLY direction as the protein-level differences but did not meet the predefined transcriptomic significance criterion after multiple-testing correction. Accordingly, the integrated findings provide a phenotype-oriented, hypothesis-generating molecular framework accompanying the higher measured IMP content in NJ rather than a validated regulatory mechanism. These findings should be interpreted in light of the non-age-matched design, the limited proteomic and transcriptomic sample sizes, and the provisional identities of the untargeted metabolomic features. In addition, because free taste-active amino acids, taste-active peptides, and sensory responses were not evaluated, the sensory significance of the higher IMP content requires independent validation.

## Figures and Tables

**Table 1:** Comparison of IMP content in the longissimus dorsi muscle between NJ and DLY pigs.

Variable	NJ (*n* = 6)	DLY (*n* = 6)	Mean Difference	95% CI of Mean Difference	Hedges’ g (95% CI)	*p* Value
IMP (mg/kg)	1965 ± 134	1407.7 ± 115.1	557.33	396.64–718.03	4.12 (2.03–6.15)	<0.0001

Values are presented as mean ± SD (*n* = 6 per group). The mean difference was calculated as NJ − DLY. The between-group difference was assessed using a two-sided independent-samples Student’s *t*-test. Hedges’ g is presented as the standardized effect size with its 95% confidence interval. CI, confidence interval; IMP, inosine 5′-monophosphate; NJ, New Jilin Black pigs; DLY, Duroc × Landrace × Yorkshire pigs.

## Data Availability

The original contributions presented in this study are included in the article/Supplementary Materials. Further inquiries can be directed to the corresponding authors.

## Supplementary Materials

The following supporting information can be downloaded at https://www.mdpi.com/article/10.3390/foods15213950/s1. Figure S1: Experimental design and proteomics-led multi-omics workflow of the study; Figure S2: Quality-control assessment of the untargeted metabolomic analysis; Figure S3: Principal component analysis of untargeted metabolomic profiles in NJ and DLY pigs; Figure S4: Exploratory partial least squares-discriminant analysis (PLS-DA) of untargeted metabolomic profiles in NJ and DLY pigs; Figure S5: Representative amino acid derivative- and small peptide-like metabolic features differing between NJ and DLY longissimus dorsi muscles; Table S1: Ingredient composition and analyzed nutrient levels of the basal diet provided to NJ and DLY pigs; Table S2: RNA quality, sequencing, and mapping statistics for samples used in transcriptomic analysis; Table S3: Complete differential expression results from the transcriptomic analysis of NJ and DLY longissimus dorsi muscle; Table S4: Complete protein-level differential-abundance statistics from the proteomic analysis of NJ and DLY longissimus dorsi muscle; Table S5: Complete differential-abundance statistics for ion-mode-specific metabolic features detected by untargeted metabolomics in NJ and DLY longissimus dorsi muscle; Table S6: Metabolic features meeting FDR and fold-change criteria in NJ and DLY longissimus dorsi muscle; Table S7: Metabolic features meeting FDR, fold-change, and VIP criteria in NJ and DLY longissimus dorsi muscle; Table S8: Statistical and identification information for representative metabolic features in NJ and DLY longissimus dorsi muscle.
