# Supplementary material for: Proteomics-Led Multi-Omics Analysis Characterizes Candidate Molecular Features Accompanying Higher IMP Content in the Longissimus dorsi Muscle of New Jilin Black Pigs
Source: Foods. 2026 Nov 3;15(21):3950. doi: 10.3390/foods15213950 (PMC13763219; doi:10.3390/foods15213950)
Supplement: Supplementary file 1 [file foods-15-03950-s001.zip › Supplementary_Materials.pdf]

# Supplementary Materials

**Manuscript title:**

**Proteomics-Led Multi-Omics Analysis Characterizes Candidate Molecular Features Accompanying Higher IMP Content in the Longissimus dorsi Muscle of New Jilin Black Pigs**

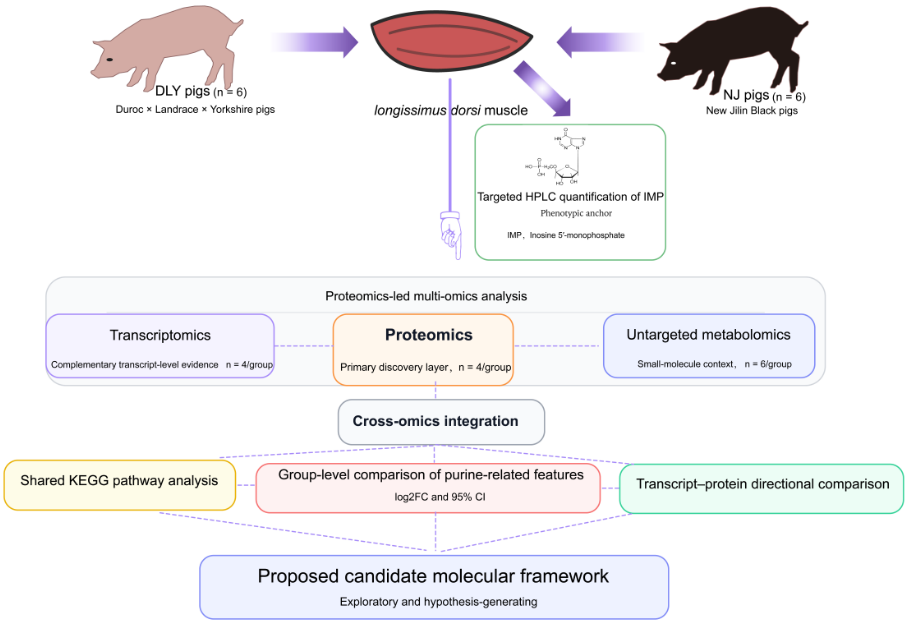


**Figure S1.** Experimental design and proteomics-led multi-omics workflow of the study. Six New Jilin Black (NJ) pigs and six Duroc × Landrace × Yorkshire (DLY) pigs were included in the study. Targeted HPLC quantification of inosine 5′-monophosphate (IMP) was performed using six biological replicates per group and served as the phenotypic anchor. Untargeted metabolomics was performed using six biological replicates per group, whereas proteomic and transcriptomic analyses were performed using four biological replicates per group, with the same four animals used for both omics layers. Proteomics was used as the primary discovery layer, untargeted metabolomics characterized the accompanying small-molecule context, and transcriptomics provided complementary transcript-level evidence. Cross-omics integration included shared KEGG pathway analysis, group-level comparison of purine-related protein and metabolite features based on $\log_2$FC estimates and 95% confidence intervals, and descriptive transcript–protein directional comparison. The integrated results were summarized in a proposed candidate molecular framework that should be interpreted as exploratory and hypothesis-generating rather than as a validated causal mechanism.

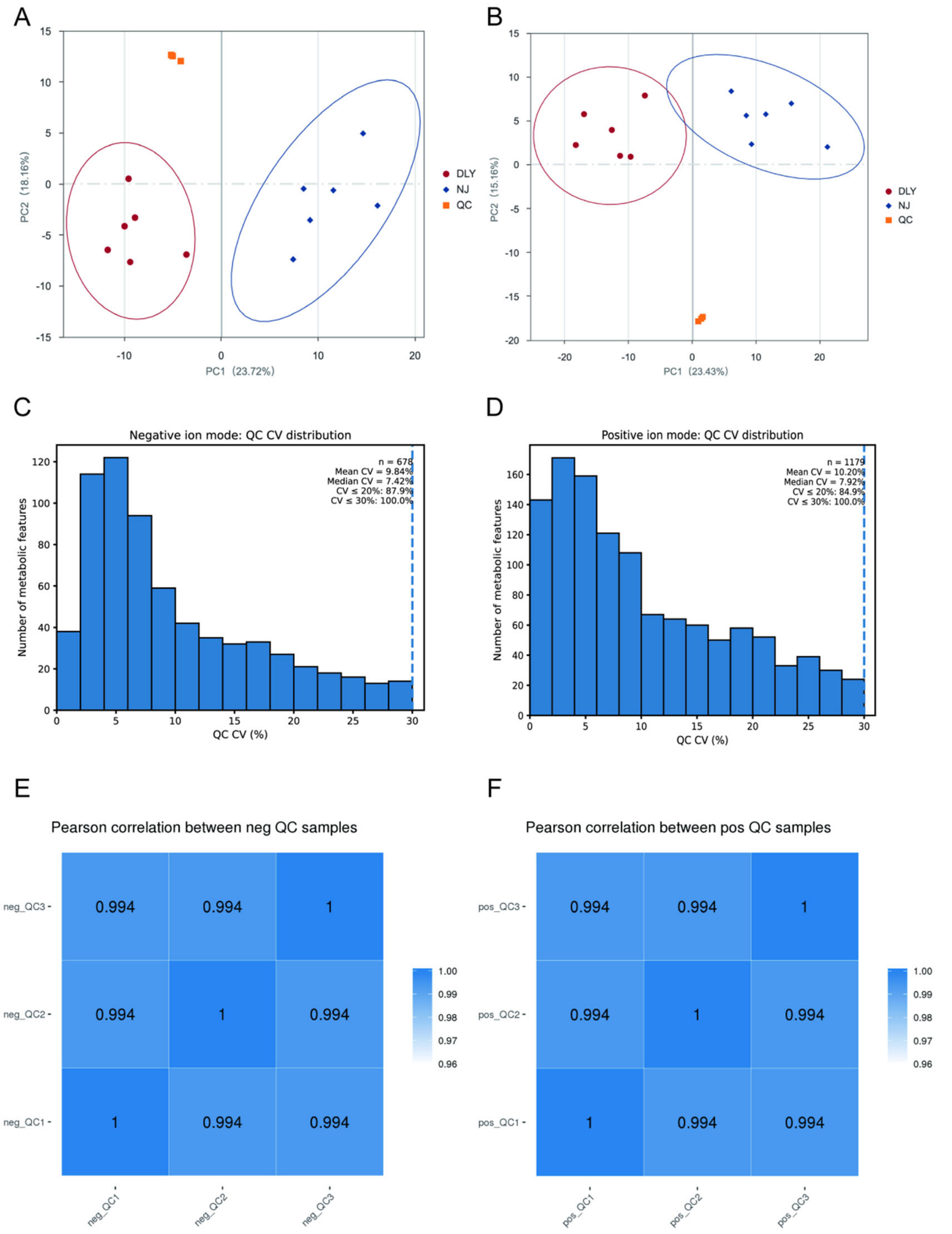


**Figure S2.** Quality-control assessment of the untargeted metabolomic analysis. (A,B) Principal component analysis (PCA) score plots of DLY and NJ samples together with pooled quality-control (QC) samples in negative- and positive-ion modes, respectively. (C,D) Distributions of QC coefficients of variation (CVs) for the retained metabolic features in negative- and positive-ion modes, respectively. The dashed line indicates a QC CV of 30%. (E,F) Pearson correlation heatmaps among pooled QC samples in negative- and positive-ion modes, respectively. The mean QC CVs of the retained metabolic features were 9.84% and 10.20% in negative- and positive-ion modes, respectively. In total, 87.9% and 84.9% of the retained features had QC CVs

≤ 20%, respectively, and all retained features had QC CVs ≤ 30%. Pairwise Pearson correlation coefficients among pooled QC samples were 0.994 in both ionization modes.

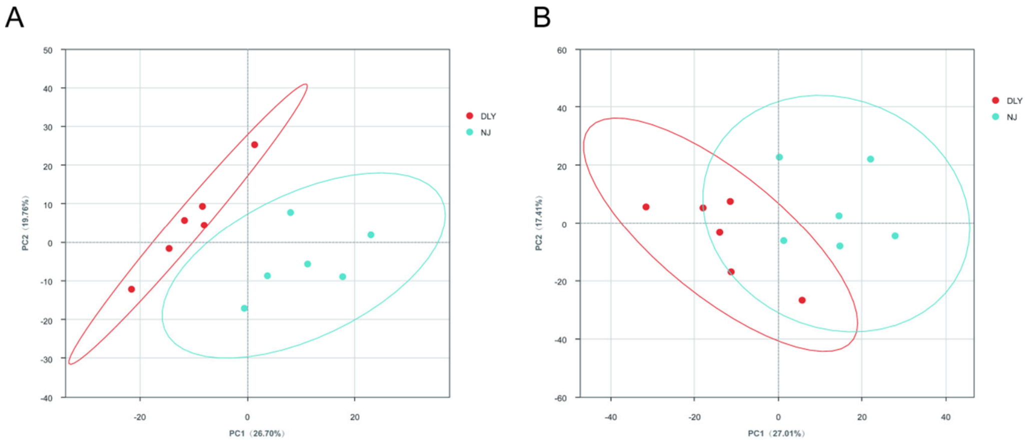


**Figure S3.** Principal component analysis of untargeted metabolomic profiles in NJ and DLY pigs. (A) PCA score plot in negative-ion mode, with PC1 and PC2 explaining 26.70% and 19.76% of the total variance, respectively. (B) PCA score plot in positive-ion mode, with PC1 and PC2 explaining 27.01% and 17.41% of the total variance, respectively. Ellipses represent the 95% confidence regions for each group. NJ, New Jilin Black pigs; DLY, Duroc × Landrace × Yorkshire pigs.

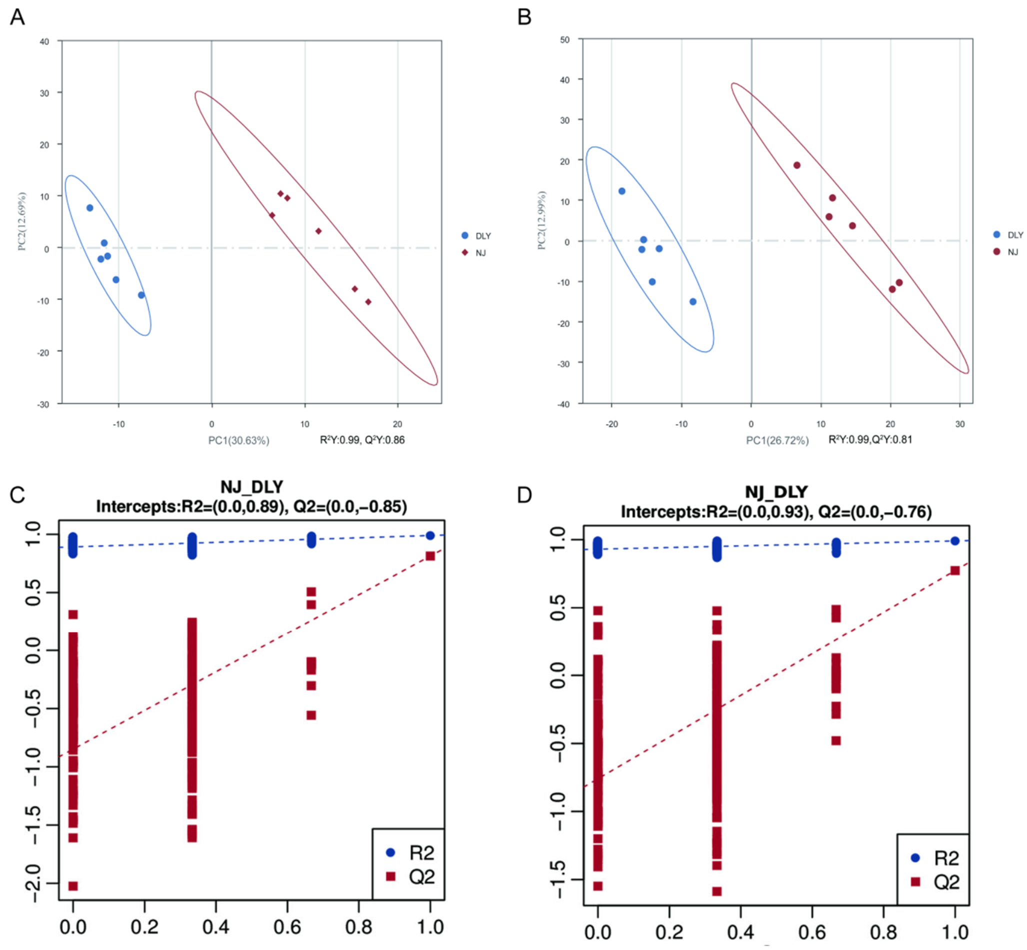


**Figure S4.** Exploratory partial least squares-discriminant analysis (PLS-DA) of untargeted metabolomic profiles in NJ and DLY pigs. (A) PLS-DA score plot for the negative-ion dataset ($R^2Y = 0.99$, $Q^2Y = 0.86$). (B) PLS-DA score plot for the positive-ion dataset ($R^2Y = 0.99$, $Q^2Y = 0.81$). Ellipses represent the 95% confidence regions for each group. (C) Corresponding 200-permutation test for the negative-ion PLS-DA model, with $R^2$ and $Q^2$ intercepts of 0.89 and −0.85, respectively. (D) Corresponding 200-permutation test for the positive-ion PLS-DA model, with $R^2$ and $Q^2$ intercepts of 0.93 and −0.76, respectively. The high R2 intercepts indicated susceptibility of the supervised models to overfitting. Accordingly, PLS-DA was treated as an exploratory supervised analysis, and VIP scores were used only as complementary multivariate information rather than as the primary criterion for defining differential metabolic features. NJ, New Jilin Black pigs; DLY, Duroc × Landrace × Yorkshire pigs; $R^2Y$, goodness of fit; $Q^2Y$, cross-validated predictive ability; VIP, variable importance in projection.

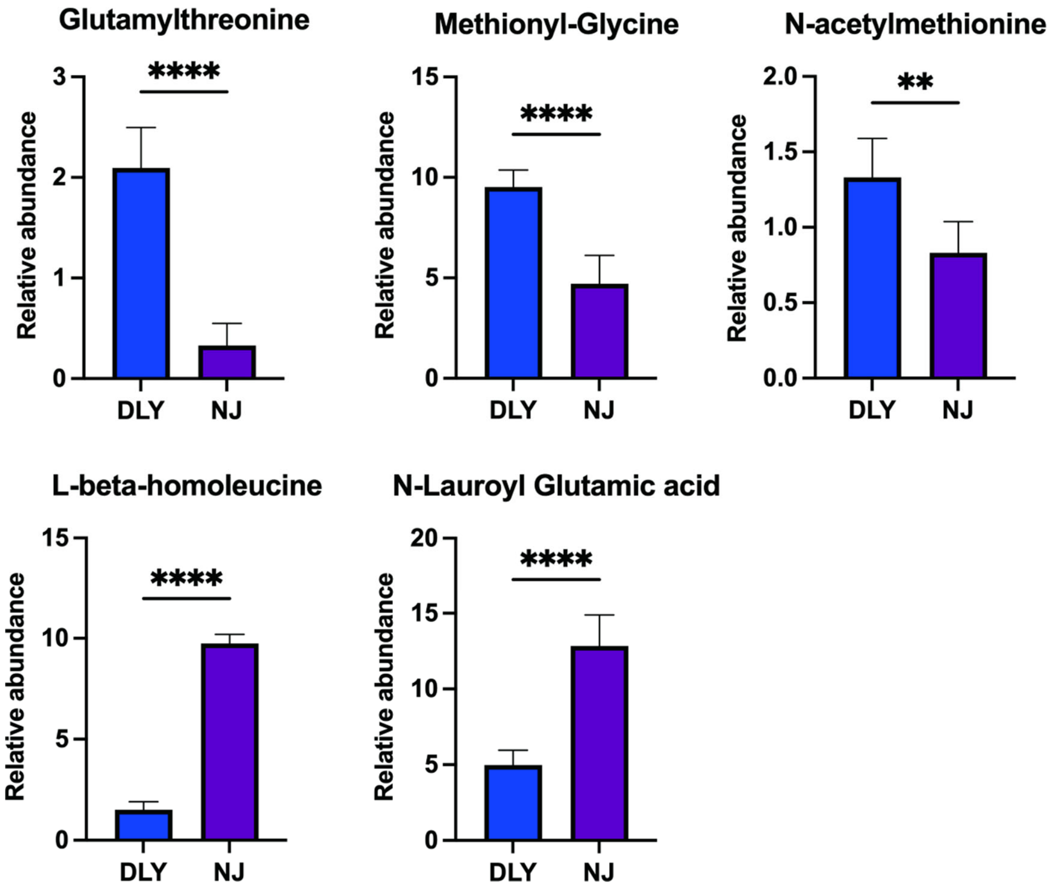


**Figure S5.** Representative amino acid derivative- and small peptide-like metabolic features differing between NJ and DLY longissimus dorsi muscles. Relative abundances of features annotated as glutamylthreonine, methionyl-glycine, N-acetylmethionine, L-beta-homoleucine, and N-lauroyl glutamic acid are presented as mean ± SD (n = 6 biological replicates per group). Statistical significance shown in the plots is based on P values obtained from the GraphPad Prism analysis: ** $P < 0.01$ and **** $P < 0.0001$. These P values are shown for visualization of the group comparisons, whereas differential-abundance significance in the untargeted metabolomic analysis was determined using BH-adjusted q values as described in the Methods. These annotated features are presented as supplementary information on broader metabolic differences between the two groups and should not be interpreted as direct measurements of free amino acid concentrations or confirmed taste-active peptides. NJ, New Jilin Black pigs; DLY, Duroc × Landrace × Yorkshire pigs.

**Table S1**. Ingredient composition and analyzed nutrient levels of the basal diet provided to NJ and DLY pigs.

| Ingredients | Content |
|---|---|
| Corn (CP, 7.06%) | 74% |
| Soybean meal (CP, 42.75%) | 17% |
| Wheat bran | 5% |
| Soybean oil | 1% |
| Dicalcium phosphate | 1% |
| Limestone | 0.5% |
| Sodium chloride | 0.3% |
| Choline chloride (50%) | 0.2% |
| Premix[1] | 1% |
| Total | 100% |
| **Analyzed nutrient levels** | |
| Digestible energy, MJ/kg | 13.65 |
| Crude protein, % | 15.06 |
| Crude fiber, % | 3.29 |
| Neutral detergent fiber, % | 14.78 |
| Acid detergent fiber, % | 4.07 |
| Calcium, % | 0.63 |
| Total phosphorus, % | 0.42 |

[1] The premix provided the following per kilogram of diet: vitamin A, 3000 IU; vitamin $D_3$, 500 IU; vitamin E, 15 IU; vitamin $K_3$, 5 mg; vitamin $B_1$, 10 mg; vitamin $B_2$, 15 mg; vitamin $B_6$, 10 mg; vitamin $B_{12}$, 20 μg; niacin, 20 mg; pantothenic acid, 24 mg; Cu, 5 mg; Fe, 100 mg; Mn, 10 mg; Zn, 100 mg; and Se, 0.3 mg.

**Table S2**. RNA quality, sequencing, and mapping statistics for samples used in transcriptomic analysis.

| Sample | RIN | Clean reads | Clean bases (Gb) | Q30 (%) | Total mapping (%) | Unique mapping (%) |
|---|---|---|---|---|---|---|
| DLY1 | 8.9 | 50784838 | 7.62 | 97.08 | 93.66 | 88.95 |
| DLY2 | 9.3 | 47770806 | 7.17 | 97.35 | 93.33 | 88.26 |
| DLY3 | 9.2 | 48709544 | 7.31 | 97.25 | 93.41 | 88.90 |
| DLY4 | 8.4 | 45867170 | 6.88 | 96.84 | 92.87 | 88.45 |
| NJ1 | 8.6 | 48621426 | 7.29 | 97.27 | 92.13 | 86.41 |
| NJ2 | 8.2 | 48743656 | 7.31 | 96.66 | 92.99 | 88.36 |
| NJ3 | 8.4 | 46906348 | 7.04 | 96.29 | 92.34 | 87.65 |
| NJ4 | 8.0 | 47134334 | 7.07 | 95.37 | 94.01 | 89.62 |

Note: RIN, RNA integrity number; Q30, percentage of bases with a Phred quality score ≥ 30; Gb, gigabases.
